# Oxidation state-specific fluorescent copper sensors reveal oncogene-driven redox changes that regulate labile copper(II) pools

**DOI:** 10.1073/pnas.2202736119

**Published:** 2022-10-17

**Authors:** Aidan T. Pezacki, Carson D. Matier, Xingxing Gu, Eric Kummelstedt, Sarah E. Bond, Laura Torrente, Kelly L. Jordan-Sciutto, Gina M. DeNicola, Timothy A. Su, Donita C. Brady, Christopher J. Chang

**Affiliations:** ^a^Department of Chemistry, University of California, Berkeley, CA 94720;; ^b^Department of Cancer Biology, Perelman School of Medicine, University of Pennsylvania, Philadelphia, PA 19104;; ^c^Department of Oral Medicine, School of Dental Medicine, University of Pennsylvania, Philadelphia, PA 19104;; ^d^Department of Cancer Physiology, H. Lee Moffitt Cancer Center and Research Institute, Tampa, FL 33612;; ^e^Department of Chemistry, University of California, Riverside, CA 92521;; ^f^Materials Science and Engineering Program, University of California, Riverside, CA 92521;; ^g^Abramson Family Cancer Research Institute, Perelman School of Medicine, University of Pennsylvania, Philadelphia, PA 19104;; ^h^Department of Molecular and Cell Biology;; ^i^Helen Wills Neuroscience Institute, University of California, Berkeley, CA 94720

**Keywords:** activity-based sensing, fluorescent copper probe, oxidative stress, transition metal signaling, cancer metabolism

## Abstract

Copper is an essential metal nutrient for life that often relies on redox cycling between Cu(I) and Cu(II) oxidation states to fulfill its physiological roles, but alterations in cellular redox status can lead to imbalances in copper homeostasis that contribute to cancer and other metalloplasias with metal-dependent disease vulnerabilities. Copper-responsive fluorescent probes offer powerful tools to study labile copper pools, but most of these reagents target Cu(I), with limited methods for monitoring Cu(II) owing to its potent fluorescence quenching properties. Here, we report an activity-based sensing strategy for turn-on, oxidation state-specific detection of Cu(II) through metal-directed acyl imidazole chemistry. Cu(II) binding to a metal and oxidation state-specific receptor that accommodates the harder Lewis acidity of Cu(II) relative to Cu(I) activates the pendant dye for reaction with proximal biological nucleophiles and concomitant metal ion release, thus avoiding fluorescence quenching. Copper-directed acyl imidazole 649 for Cu(II) (CD649.2) provides foundational information on the existence and regulation of labile Cu(II) pools, including identifying divalent metal transporter 1 (DMT1) as a Cu(II) importer, labile Cu(II) increases in response to oxidative stress induced by depleting total glutathione levels, and reciprocal increases in labile Cu(II) accompanied by decreases in labile Cu(I) induced by oncogenic mutations that promote oxidative stress.

## Significance

Copper sustains fundamental chemical processes across all kingdoms of life by cycling between two major oxidation states, Cu(I) and Cu(II). However, in contrast to advances in fluorescent sensors and related probes to help decipher Cu(I) biology, Cu(II) detection remains lacking. We present a strategy using metal-directed acyl imidazole chemistry to enable both metal and oxidation state-specific Cu(II) detection by a tandem activity-based sensing/labeling reaction. We use this platform to identify the existence of loosely bound, labile Cu(II) pools, a Cu(II) import protein in cells, and reciprocal Cu(II) elevations and Cu(I) deficiencies driven by a loss of antioxidant defense in cellular models of cancer.

Copper is an essential nutrient for life (1), where its oxidation reduction (redox) properties are used in catalytic roles to support biological processes such as oxygen transport, metabolism, respiration, growth, and proliferation by cycling between Cu(I) and Cu(II) oxidation states (2–6). However, misregulation of copper-dependent redox activity can also cause aberrant production of reactive oxygen species (ROS) (7), and this accompanying oxidative stress contributes to cancer (4), neurodegenerative diseases (3, 8, 9), inflammation and immune response (10, 11), and genetic copper metabolism disorders (12, 13). Biological copper is partitioned into two main pools: static pools that are tightly bound within metalloproteins, largely as cofactors in active sites, and dynamic labile pools that are weakly bound to low molecular weight ligands and undergo rapid intercellular and intracellular transport (14–19). The physiological significance of labile copper has become increasingly recognized with the discovery of its contributions to a broad array of signaling pathways (20, 21), including work from our laboratory on copper-dependent transition metal signaling in lipolysis (22), spontaneous and stimulated neuronal activity (19, 22–24), and sleep–wake cycles (25). Reversible binding of copper at allosteric sites to regulate protein function has given rise to the concept of metalloallostery (20, 21). Additionally, labile copper has been linked to proproliferative and autophagic kinase signaling pathways involved in oncogenesis (26–28), with the term *cuproplasia* being recently coined as copper-dependent cell growth and proliferation in disease (4).

Despite the fact that both Cu(I) and Cu(II) are viable oxidation states for this metal nutrient under physiological and pathological conditions, cellular studies of labile copper pools have largely focused on Cu(I), as the conventional view is that the reducing environment of the cell favors this oxidation state. Along these lines, the existence and characterization of labile Cu(II) pools have been insufficiently studied, largely due to a lack of methods for detecting Cu(II) and distinguishing it from Cu(I) in the cellular milieu, with specificity for both metal and oxidation state. Indeed, relative to advances in Cu(I) fluorescence detection (3, 15–18, 21, 23–25, 27, 29–43), Cu(II)-sensitive fluorescent sensors have had limited biological application, as this paramagnetic ion is a potent fluorescence quencher by electron or energy transfer pathways and often leads to turn-off responses that limit the spatial resolution of synthetic fluorophores (15, 44). Therefore, the range of techniques that are available for studying Cu(II) are largely restricted to indirect or low-throughput techniques like spectrophotometry-based copper reduction assays (45) and electrophysiology (46).

To meet the need for detection of intracellular labile Cu(II) pools and their response to external and internal stimuli, we reasoned that an activity-based sensing approach might be amenable to solve this challenge, as analyte detection is often coupled to bond-breaking reactions that dissociate the analyte from the dye reporter (47, 48). We thus turned our attention to an activity-based sensing approach to monitor copper in live cells by using copper-directed acyl imidazole (CDAI) chemistry (22, 49). In this design, binding of a Lewis acidic copper ion increases the electrophilicity of the acyl imidazole unit to trigger nucleophilic addition by proximal amino acids, enabling the detection of localized copper hotspots while preventing probe diffusion. Indeed, the tandem sensing and labeling process occurs with concomitant metal release, which minimizes potential metal dye quenching pathways. While this first-generation design established that CDAI chemistry can achieve metal specificity, a major limitation is a lack of oxidation state specificity, as the probe responds both to Cu(I) and Cu(II). We now present the design, synthesis, and biological applications of Cu(II)-specific CDAI probes. We apply hard–soft acid–base considerations to achieve Cu(II) specificity, where judicious replacement of one of the thioether ligands in the metal receptor of the original CDAI probe with a harder carboxylate ligand can better accommodate the harder Lewis acidity of Cu(II) relative to Cu(I) (50). The red-emissive version, copper-directed acyl imidazole 649 for Cu(II), **CD649.2**, exhibits high metal and oxidation state specificity. This probe enables visualization of labile Cu(II) pools in live cells as well as dynamic changes in these pools upon exogenous copper supplementation or depletion, establishing the existence of a mobile intracellular Cu(II) pool. We use this probe to identify an ion channel for Cu(II) uptake, expansion of Cu(II) pools caused by oxidative stress, and reciprocal Cu(II) increases and Cu(I) decreases triggered by introduction of oncogenes. By establishing the existence of a labile intracellular Cu(II) pool and its response to cellular redox status, this work provides a starting point for broader investigations of transition metal signaling where these unique elements can achieve an additional layer of biochemical regulation in the form of redox cycling that goes beyond spatial and temporal control afforded by traditional inorganic calcium, potassium, sodium, and chloride signals.

## Results and Discussion

### Design, Synthesis, and Characterization of CDAI Probes with Cu(II) Selectivity.

**Scheme 1.**
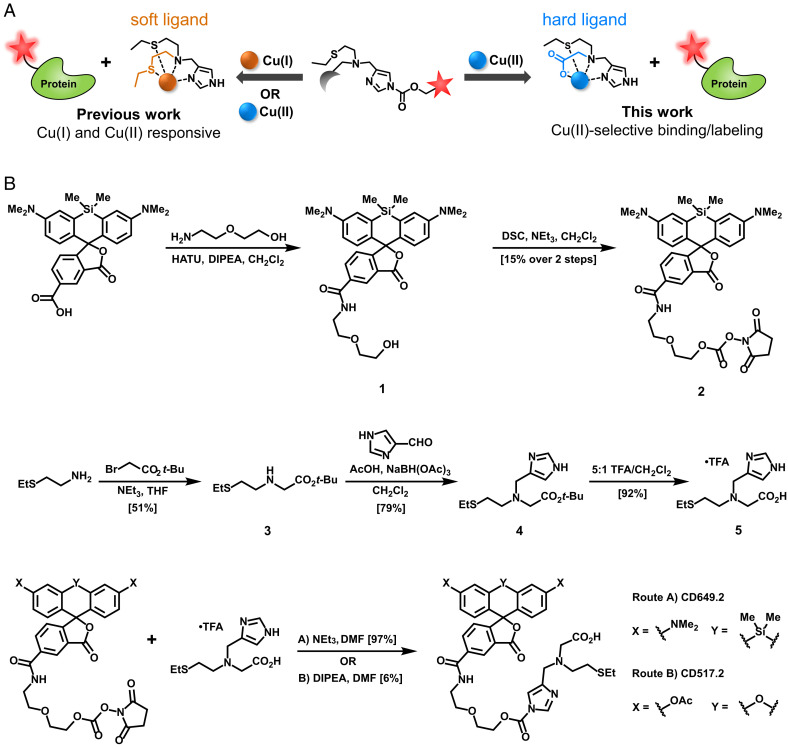
(*A*) Design strategy for tuning hard–soft acid–base properties of a metal receptor in a CDAI to increase oxidation state selectivity for Cu(II) over Cu(I). Modular substitution of a soft thioether donor with a harder carboxylate ligand affords greater Cu(II) selectivity. (*B*) Synthesis of the Cu(II)-selective CDAI probes **CD649.2** and **CD517.2**.

The metal-directed acyl imidazole strategy is well suited to paramagnetic Cu(II) detection. Activity-based sensing is achieved by metal binding, triggering Lewis acid activation of the acyl imidazole unit for trapping onto proximal biological nucleophiles to preserve spatial resolution of the dye while simultaneously releasing the fluorescence quenching Cu(II) ion analyte. We designed dual metal and oxidation state selectivity into the CDAI platform by tuning the hard–soft acid–base pairing of the metal chelating portion of the probe (Scheme 1*A*). Cu(II) is a harder Lewis acid compared to Cu(I) and therefore has a preference for harder Lewis bases. Meanwhile, Zn(II) is a biologically relevant transition metal with similar acidic properties to Cu(II) (51); however, it prefers triamine and tetramine-based ligand scaffolds (15). We hypothesized that replacement of one of the soft thioether ligands with a hard carboxylate ligand would bias selectivity toward Cu(II) over Cu(I) without affecting overall metal selectivity (50). To this end, we synthesized two CDAI for Cu(II) dyes, the red **CD649.2** (52) and green **CD517.2** (Scheme 1*B* and *SI Appendix*, Scheme S1).

**Fig. 1.**
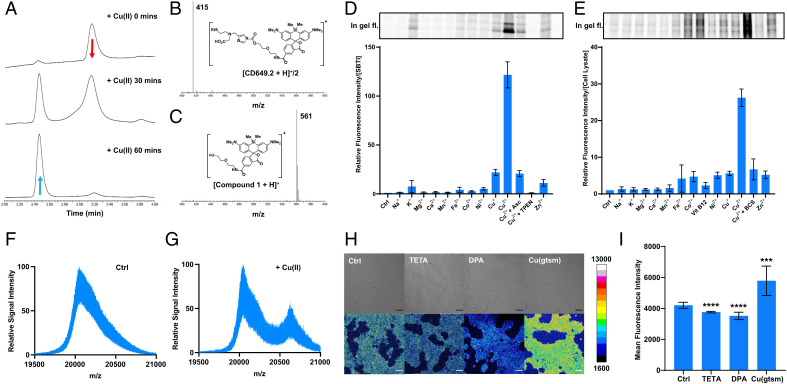
**CD649.2** is a Cu(II)-responsive, activity-based sensing fluorescent probe with metal and oxidation state selectivity and is capable of detecting changes in labile intracellular Cu(II) pools by live-cell confocal microscopy. (*A*) Liquid chromatography chromatograms of the reaction between 100 μM **CD649.2** and 250 μM CuSO_4_ in PBS for 1 h, showing complete conversion of **CD649.2** to compound **1**. (*B*) The mass of intact **CD649.2** was detected at a retention time of 3.2 min. (*C*) The hydrolyzed product of **CD649.2** and Cu(II) is detected at a retention time of 2.5 min. In-gel fluorescence images and integrated fluorescence intensities of **CD649.2** incubated with (*D*) SBTI as a model protein or (*E*) HEK 293T cell lysates. Samples were preincubated with biologically relevant metal ions for 5 min (s-block metal ions at 1 mM, d-block metal ions at 10 μM, TPEN at 50 μM, ascorbate at 30 µM), followed by incubation with **CD649.2** at concentrations of 10 μM (SBTI) or 30 μM (cell lysates), at room temperature for 30 min. In-gel fluorescence for sodium dodecyl sulfate–polyacrylamide gel electrophoresis was scanned by ChemiDoc MP, and signal intensity was analyzed by Image Lab. Signal intensity was normalized to protein concentration as measured by Pierce Silver Stain Kit. Mass spectra of SBTI treated with (*F*) **CD649.2** alone or (*G*) **CD649.2** and Cu(II). SBTI (5 mg/mL) was preincubated with solvent vehicle control or 100 μM CuSO_4_ for 5 min followed by incubation with 50 μM **CD649.2** at room temperature for 30 min. The mass adduct of SBTI (20.1 kDa) + conjugated probe (603.75 Da) was detected for Cu(II)-treated protein only. Spectra were obtained via matrix-assisted laser desorption/ionization time-of-flight mass spectrometry (matrix: 10 mg/mL sinapinic acid in 50% MeCN/50% H_2_O/0.1% TFA). (*H*) Confocal fluorescence microscopy images of HEK 293T cells treated with solvent vehicle control, chelators TETA or DPA for 24 h, or the ionophore Cu(gtsm) for 1 h. Cells were washed twice with Hanks' Balanced Salt Solution (HBSS), incubated with **CD649.2** for 1 h, washed once with HBSS, and then imaged. (*I*) Normalized cellular fluorescence intensities of the HEK 293T cells as determined in ImageJ, showing an increase in fluorescence signal when treated with Cu(gtsm) and a decrease in signal in response to TETA or DPA. Fluorescence intensity of **CD649.2** was determined from experiments performed in triplicate with λex = 633 nm. Error bars denote SD (*n* = 12). Scale bar = 50 μm. *P < 0.05, **P < 0.01, ***P < 0.001, and ****P < 0.0001.

To determine the Cu(II) binding affinity of the metal receptor, we titrated compound **5** (**NSCO_2_**) to a solution of 1 mM Tris(2-pyridylmethyl)amine and 1 mM of Cu(II) (*SI Appendix*, Fig. S1). A *K_d_* value was obtained by recording changes in the ultraviolet–visible absorption spectra of the copper-bound Tris(2-pyridylmethyl)amine whose Cu(II) affinity is known (53), providing an estimated *K_d_* of 1.93 × 10^−14^ M for **NSCO_2_**, calculated via previously reported methods (54). To evaluate the reactivity of the Cu(II)-selective CDAI probes, we monitored the hydrolysis reaction of **CD649.2** in the presence of Cu(II) via liquid chromatography–mass spectrometry (Fig. 1*A*). Liquid chromatograms of the reaction between 100 μM **CD649.2** and 2.5 equivalence of Cu(II) in phosphate-buffered saline (PBS) showed complete conversion of the probe (415 *m*/*z*, Fig. 1*B*) within 1 h, affording a product with a mass of 561 *m*/*z* (Fig. 1*C*), corresponding to the hydrolyzed **CD649.2** species with loss of the copper receptor. The reaction between 100 μM **CD517.2** and 2.5 equivalence of Cu(II) in PBS similarly showed complete conversion of the probe (409 *m*/*z*) within 1 h, affording a product with a mass of 548 *m*/*z*, corresponding to the hydrolyzed product (*SI Appendix*, Fig. S2). In order to assess the ability of **CD649.2** to respond to Cu(II) by Lewis acid activation of its acyl imidazole unit for proximal protein labeling, we performed in vitro protein labeling assays on whole cell lysates incubated with **CD649.2** and varying concentrations of Cu(II). In-gel analysis of protein solutions established that **CD649.2** was capable of labeling proteins in HEK 293T cell lysates in a Cu(II)-responsive dose-dependent manner, with maximal labeling occurring with a 1:1 ratio of probe and metal (*SI Appendix*, Fig. S3). We then tested the metal and oxidation state selectivity of **CD649.2** by using soybean trypsin inhibitor (SBTI) as a model protein. SBTI has multiple surface-accessible amino acids with nucleophilic side chains (55) that could undergo facile reaction with the CDAI probe. The data show that **CD649.2** is highly selective for copper over a range of biologically relevant metals, with about sixfold selectivity for Cu(II) over Cu(I) (Fig. 1*D* and *SI Appendix*, Fig. S4), which is also confirmed in cell lysate models (Fig. 1*E* and *SI Appendix*, Fig. S5). The result is a marked improvement in oxidation state selectivity over the first-generation probe **CD649** (*SI Appendix*, Fig. S6), further corroborated by treating cell lysate with **NSCO_2_**, which inhibits protein labeling by **CD649** through competitive chelation of Cu(II) (*SI Appendix*, Fig. S7). Analogous experiments with **CD517.2** showed a similar preference for Cu(II) (*SI Appendix*, Fig. S8). We confirmed that the observed fluorescence signal arises from labeled protein via mass spectrometry analysis on SBTI and **CD649.2**. In the absence of Cu(II), only unlabeled SBTI (20.1 kDa) was detected (Fig. 1*F*). Upon addition of 100 μM Cu(II), a mass corresponding to SBTI + the fluorophore and linker (20.6 kDa) emerges (Fig. 1*G*). Similarly, unlabeled SBTI was detected upon addition of 50–250 μM Cu(I) and 50 μM **CD649.2**, whereas addition of 50–250 μM Cu(II) and 50 μM **CD649.2** resulted in increasing intensity of the labeled peak for increasing Cu(II) concentration (*SI Appendix*, Fig. S9).

### CD517.2 and CD649.2 Enable Imaging of Labile Cu(II) Pools in Living Cells.

With these probes in hand, we sought to evaluate the ability of **CD649.2** to identify and detect changes in labile Cu(II) pools in living cells in response to exogenous copper supplementation and endogenous copper chelation. HEK 293T cells were treated with the Cu(II) ionophore Cu(gtsm) as a copper supplement or with the Cu(II)-selective chelators triethylenetetramine (TETA) or D-penicillamine (DPA) (Fig. 1 *C* and *D*), which showed that the probe can identify labile Cu(II) pools and expansion and depletion of these pools by these various treatments. Interestingly, although TETA has a higher affinity for Cu(II) (56), DPA-treated cells showed a slightly lower **CD649.2** fluorescence signal, which could result from DPA-mediated reduction of Cu(II) to Cu(I) in addition to Cu(II) chelation (57). To confirm that the observed reduction in fluorescence is a result of sequestered labile Cu(II), we sequentially treated HEK293T cells with TETA or DPA followed by Cu(gtsm) (*SI Appendix*, Fig. S10). HEK293T cells treated with 200 μM TETA or DPA for 24 h and 2 μM Cu(gtsm) for 1 h showed a slight decrease in **CD649.2** fluorescence signal versus vehicle control, indicating that the exogenously added Cu(II) was sequestered by the excess chelator present. Additionally, we treated HEK293T cells with varying concentrations of Cu(II) chelator to determine whether endogenous labile Cu(II) levels could be further depleted **(***SI Appendix*, Fig. S11**)**. While we do see a reduction in **CD649.2** fluorescence upon treatment with either TETA or DPA, this reduction is not dependent on chelator concentration in the range of 200–500 µM, suggesting that a majority of the bioavailable labile Cu(II) has been depleted. Dual-color imaging experiments with a Hoechst 33342 nuclear stain confirm that **CD649.2** covalently labels biomolecules in both the cytosol and the nucleus in this cell line (*SI Appendix*, Fig. S12) and maintains high cell viability as measured by a PrestoBlue assay (*SI Appendix*, Fig. S13). Imaging experiments using **CD517.2** showed similar changes in Cu(II)-dependent fluorescence upon treatment with copper supplements or chelators (*SI Appendix*, Fig. S14). Taken together, these CDAI probes identify pools of labile Cu(II) in cells, adding this oxidation state as a source of bioavailable copper beyond Cu(I).

### CD649.2 Identifies Divalent Metal Transporter 1 as an Oxidation State-Specific Cu(II) Importer.

**Fig. 2.**
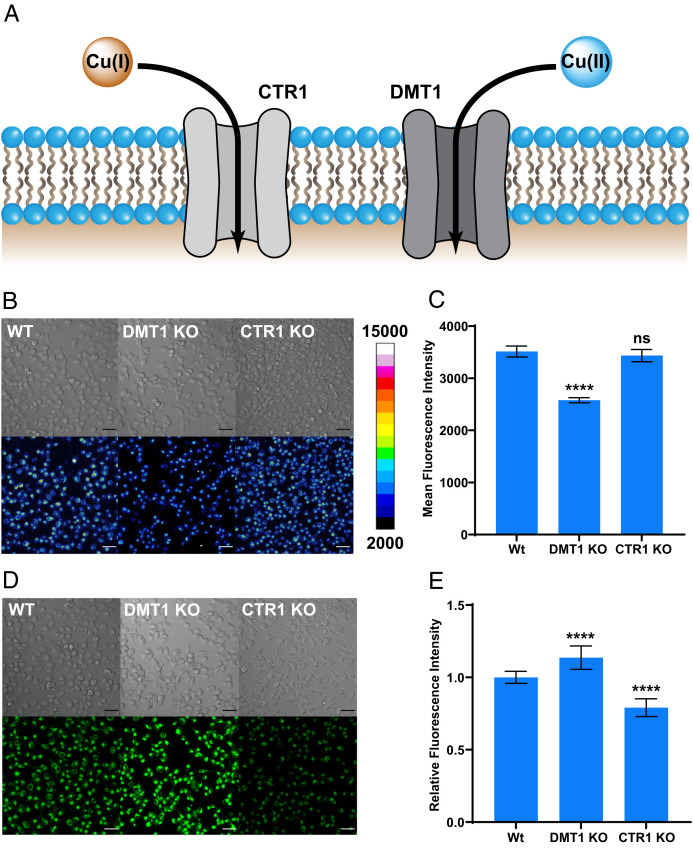
Live-cell imaging with **CD649.2** identifies DMT1 as a copper transporter with selectivity for Cu(II), while the Cu(I)-specific fluorescent sensor **CF4** identifies CTR1 but not DMT1 as a Cu(I)-selective importer. (*A*) Model for oxidation state-selective copper nutrient uptake pathways, where CTR1 mediates Cu(I) uptake and DMT1 mediates Cu(II) uptake. (*B*) Confocal fluorescence microscopy images comparing WT H1299 cells with genetic knockout of DMT1 (DMT1 KO) or CTR1 (CTR1 KO). Cells were then washed once with HBSS, incubated with **CD649.2** for 30 min, washed once with HBSS, and then imaged. (*C*) Average cellular fluorescence intensity of **CD649.2** determined in ImageJ, showing a decrease in fluorescence signal for DMT1 KO but no significant changes for CTR1 KO. (*D*) Confocal fluorescence microscopy images of H1299 WT, DMT1 KO, and CTR1 KO cells stained with **CF4**. Cells were washed once with HBSS (+Ca, Mg), incubated with 1 µM **CF4** in HBSS (+Ca, Mg) for 30 min, and then imaged. (*E*) Average cellular fluorescence intensity of **CF4** determined in ImageJ, showing a decrease in fluorescence signal for CTR1 KO versus WT and a slight increase in signal for DMT1 KO versus WT. Fluorescence intensity of **CD649.2** and **CF4** was determined from experiments performed in triplicate with λex = 633 nm for **CD649.2** and λex = 488 nm for CF4. Error bars denote SD (*n* = 12). Scale bar = 50 μm. *P < 0.05, **P < 0.01, ***P < 0.001, and ****P < 0.0001; ns, not statistically significant.

With data establishing the existence of labile intracellular Cu(II) pools that can respond to copper supplementation or chelation, we next sought to elucidate foundational principles of its biological regulation. In this context, we hypothesized that copper uptake is dependent on oxidation state, where divalent metal transporter 1 (DMT1) could serve as a Cu(II) importer, akin to the known role of copper transporter 1 (CTR1) as a Cu(I) importer (Fig. 2*A*). Indeed, DMT1 is responsible for Fe(II) gastrointestinal and endosomal uptake and can mediate the uptake of multiple divalent metals (46). However, Cu(II) import by DMT1 remains an open question (58), as previous studies to address this issue have been limited to indirect electrophysiological and spectrophotometry-based methods (59). To provide direct evidence for the potential role of DMT1 in Cu(II) transport, we used **CD649.2** to measure labile Cu(II) accumulation in mammalian cell models with genetic knockout of either DMT1 or CTR1 (60). DMT1 and CTR1 knockout H1299 cell lines (DMT1 KO and CTR1 KO) were created via CRISPR-Cas9 methods (59). **CD649.2** imaging reveals that DMT1 KO cells show lower levels of fluorescence compared to wild-type (WT) cells (Fig. 2 *B* and *C*). The magnitude of this decrease in **CD649.2** fluorescence is comparable to what is observed upon treatment with copper chelators TETA or DPA (*SI Appendix*, Fig. S15). In contrast, **CD649.2** imaging shows similar fluorescence intensities in CTR1 KO cells relative to WT, corroborating the selectivity for Cu(I) over Cu(II) import by CTR1. Imaging H1299 cells with **CD517.2** shows a similar pattern in fluorescence reduction for DMT1 KO cells versus WT (*SI Appendix*, Fig. S16) and TETA- or DPA-treated cells versus nontreated (*SI Appendix*, Fig. S17). Moreover, we confirmed the Cu(I) specificity of CTR1 by using the Cu(I)-selective fluorescent probe Copper Fluor-4 (**CF4**) (25), which showed the expected decrease in fluorescence signal in CTR1 KO cells relative to WT and a modest increase in DMT1 KO cells (Fig. 2 *D* and *E*). Interestingly, we found significant mitochondrial localization of **CD649.2** fluorescence signal in this cell line as determined from costaining experiments with MitoTracker Green while analogous costaining with **CD517.2** and MitoTracker Red reveals uniform labeling for the green-shifted CDAI (*SI Appendix*, Fig. S18). We propose that **CD649.2** and **C517.2** provide complementary information on labile Cu(II) status, as one can report on subcellular localization while the other reports on whole cell labile Cu(II). Collectively, these data provide direct evidence that DMT1 can deliver Cu(II) into cells and modulate the intracellular labile Cu(II) pools.

### CD649.2 Establishes a Relationship Between Increases in Cellular Oxidative Stress and Dynamic Expansion of Labile Cu(II) Pools.

**Fig. 3.**
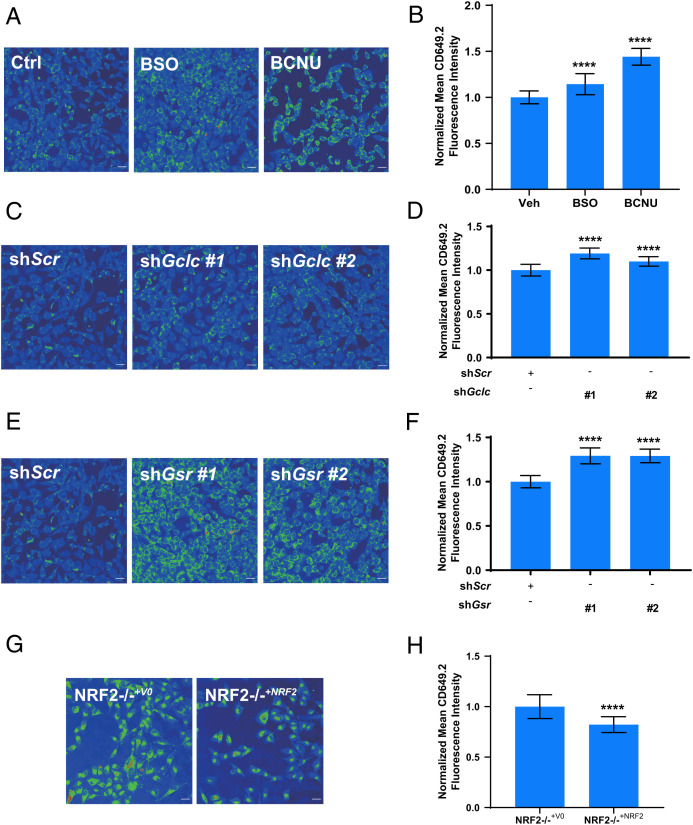
Live-cell imaging with **CD649.2** reveals elevated levels of labile Cu(II) pools induced by pharmacological or genetic manipulations of cellular redox status, including decreases in GSH production, lowering of reduced to oxidized GSH ratios (GSH/GSSG), or loss of the antioxidant regulator NRF2. (*A*) Confocal fluorescence images of MEF cells treated with solvent vehicle control, BSO, or BCNU for 4 h. Cells were washed twice with HBSS, incubated with **CD649.2** for 1 h, washed with HBSS, and then imaged. (*B*) Normalized mean **CD649.2** fluorescence intensity of MEF cells showing an increase in signal for both BSO- and BCNU-treated cells. Error bars denote SD (*n* = 30). (*C*, *E*) Confocal fluorescence images of MEF cells stably expressing nontargeting control shRNA (*Scr*), G*clc* shRNA, or G*sr* shRNA. ShRNA knockdowns were performed in duplicate, which we define as #1 and #2. Cells were washed twice with HBSS, incubated with **CD649.2** for 1 h, washed with HBSS, and then imaged. (*D*, *F*) Normalized mean **CD649.2** fluorescence intensity of MEF cells showing an increase in signal for both G*clc* shRNA and G*sr* shRNA versus *Scr* MEFs. Error bars denote SD (*n* = 30). (*G*) Confocal fluorescence microscopy images of A549 cells stably expressing NRF2^−/−+V0^ or NRF2^−/−+NRF2^. Cells were washed twice with HBSS, incubated with **CD649.2** for 1 h, washed with HBSS, and then imaged. (*H*) Normalized mean **CD649.2** fluorescence intensity of A549 cells showing a decrease in signal for NRF2^−/−+NRF2^ versus NRF2^−/−+V0^. Error bars denote SD (*n* = 30). Fluorescence intensity of **CD649.2** was determined from experiments performed in triplicate with λex = 649 nm. Scale bar = 30 μm. *P < 0.05, **P < 0.01, ***P < 0.001, and ****P < 0.0001; ns, not statistically significant.

The ability of **CD649.2** to monitor labile intracellular Cu(II) pools in an oxidation state-specific manner led us to investigate how these stores are affected by cellular redox mediators. In this context, glutathione (GSH) is a highly abundant cellular antioxidant that is critical for the maintenance of cellular redox status in equilibrium with its oxidized glutathione disulfide (GSSG) form (61). Indeed, we have recently shown that it plays a role in maintaining labile Cu(I) bioavailability (27). We first perturbed GSH homeostasis by using two different pharmacological treatments and tested their effects on labile Cu(II) pools by **CD649.2** imaging. Buthionine sulfoximine (BSO) inhibits glutamate cysteine ligase (62), the enzyme involved in the rate-determining step of GSH biosynthesis, while bis-chloroethylnitrosourea (BCNU) inhibits glutathione reductase (63), the enzyme responsible for reducing GSSG. We monitored the impact of these inhibitors on labile intracellular Cu(II) levels. We observed that both treatments gave rise to an increase in labile Cu(II) as measured by **CD649.2** imaging (Fig. 3 *A* and *B*). These data could be interpreted as a decrease in Cu(II) buffering by GSH or a more oxidizing environment that favors Cu(II). We also tested genetic manipulation of these same targets in mouse embryonic fibroblast (MEF) cells via short hairpin RNA (shRNA)-mediated knockdown of the gene encoding glutathione reductase (*Gsr*) or the catalytic subunit of glutamate cysteine ligase (*Gclc*). Knockdown of these genes produces genetic models equivalent to treatment with BCNU or BSO, respectively. We previously showed that sh*Gclc* MEFs have decreased GSH levels, sh*Gsr* MEFs have a decreased GSH/GSSG ratio, and both models have unchanged levels of total copper (27). We observed that labile Cu(II) levels, as measured by **CD649.2** for both *shGsr* and *shGclc* MEF cells, were elevated relative to cells treated with a control shRNA with a scrambled sequence (*Scr*) (Fig. 3 *C*–*F* and *SI Appendix*, Fig. S19), in agreement with the pharmacological inhibition results. In addition, we performed shRNA-mediated knockdown on the light-chain subunit of the cystine/glutamate antiporter (*xCT*) in MEFs, the subunit responsible for cystine transport and a key promoter of GSH biosynthesis (64). Labile Cu(II) and Cu(I) levels in sh*xCT* MEFs remained unchanged, as measured by **CD649.2** and **CF4**, respectively (*SI Appendix*, Figs. S20–S22). Consistent with these results, sh*xCT* MEFs showed unchanged levels of total GSH and an unaffected GSH/GSSG ratio (*SI Appendix*, Fig. S23). Thus, it is likely that the 50% reduction in *xCT* expression was not sufficient to reduce GSH status, and compensatory cystine transporter mechanisms maintain GSH levels so that copper bioavailability is unchanged. As a second model to evaluate how broader changes in cellular antioxidant status can affect labile intracellular Cu(II) homeostasis, we also evaluated the effect of loss of nuclear factor–erythroid factor 2–related factor 2 (NRF2), which up-regulates the expression of a series of antioxidant response element genes in response to oxidative stress, including those associated with GSH (65). To evaluate the impact of NRF2 on labile copper levels, we used the non–small cell lung cancer cell A549, which harbors an inactivating mutation in the NRF2 negative regulator KEAP1, in which endogenous *NRF2* was ablated via CRISPR-Cas9 (NRF2^−/−+V0^) and subsequently restored (NRF2^−/−+NRF2^) (66) and measured labile intracellular Cu(II) and Cu(I) levels by using **CD649.2** and **CF4** probes, respectively. We found a marked increase in both labile Cu(I) and labile Cu(II) pools in NRF2^−/−+V0^ cells compared with NRF2^−/−+NRF2^ control cells (Fig. 3 *G* and *H* and *SI Appendix*, Fig. S24). The observation of higher levels of both forms of labile copper in the NRF2 KO cell line relative to the rescue suggest that NRF2 contributes to the regulation of overall labile copper bioavailability rather than an oxidation state-specific effect. Elevated levels of labile copper would be expected to exacerbate oxidative stress since copper can generate ROS from both Cu(I) and Cu(II) (4). Taken together, these data establish that endogenous cellular antioxidants, acting as binders or buffers for intracellular copper, play important roles in protection against oxidative stress. As was observed for labile Cu(I) pools, cellular redox status is connected to controlling labile Cu(II) bioavailability, directly linking GSH metabolism and broad antioxidant expression to this metal nutrient in both oxidation states.

### Oncogenic BRAF^V600E^ and KRAS^G12D^ Mutations Promote Redox Stress with Reciprocal Increases in Labile Intracellular Cu(II) Pools and Decreases in Labile Intracellular Cu(I) Pools.

**Fig. 4.**
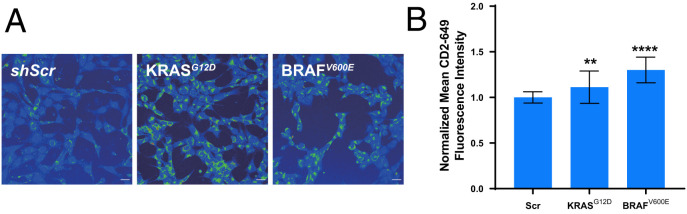
Oncogenic BRAF^V600E^ and KRAS^G12D^ lead to increases in labile Cu(II) pools with reciprocal decreases in labile Cu(I) pools. (*A*) Confocal fluorescence images of **CD649.2** in MEF cells stably expressing *Scr*, BRAF^V600E^ complementary DNA, or KRAS^G12D^ complementary DNA. Cells were washed twice with HBSS, incubated with **CD649.2** for 1 h, washed with HBSS, and then imaged. (*B*) Normalized mean **CD649.2** fluorescence intensity of MEF cells showing an increase in signal for both BRAF^V600E^ and KRAS^G12D^ mutations over *Scr*. Error bars denote SD (*n* = 30). Fluorescence intensity of **CD649.2** was determined from experiments performed in triplicate with λex = 649 nm. Scale bar = 30 μm. *P < 0.05, **P < 0.01, ***P < 0.001, and ****P < 0.0001; ns, not statistically significant.

With data establishing the connection between intracellular labile copper and redox status, we sought to further probe these relationships in a disease-relevant context. To this end, we turned our attention to the growing connection between copper and cancer (4, 26–28, 67–72), particularly with regard to cuproplasia, a copper-dependent form of cell proliferation that provides a biochemical basis for this metal nutrient as a disease vulnerability. Indeed, cancer cells exhibit an increased rate of proliferation (4), a process that requires copper for its catalytic role in driving cellular respiration via cytochrome *c* oxidase. Based on these considerations, we anticipated that Cu(II) could be favored over Cu(I) in a more oxidizing intracellular environment, and thus detection of this more oxidized form of the metal nutrient could be used as a proxy biomarker for oxidative stress in cancer. Indeed, many tumor types have an elevated metabolic demand owing to the rapid proliferation associated with tumorigenesis (4), and in response to these higher energy needs, many tumors also produce increased levels of ROS (73), which in turn should increase the pools of oxidized chemical species in the cell, including Cu(II). To explore this connection, we investigated the effects of introducing oncogenic mutations in BRAF (V600E) or KRAS (G21D) on labile intracellular Cu(II) levels in MEF model of cellular transformation. We found a marked increase in **CD649.2** fluorescence signals in both tumorigenic cell lines when compared to *Scr* controls, suggesting elevations in labile intracellular Cu(II) pools (Fig. 4 *A* and *B*). Interestingly, these data complement our laboratories’ previous report showing that these same oncogenic transformations promote decreases in labile intracellular Cu(I) pools due to lowered *Gsr* expression and increased ROS generation, with levels of total copper remaining unchanged (27). Indeed, these results indicate a unique and dynamic shift in labile Cu(I)/Cu(II) ratios that correlate to the more oxidizing cellular environment promoted by oncogenesis.

## Concluding Remarks

We have presented the design, synthesis, and evaluation of a strategy for turn-on fluorescence detection of Cu(II) with unique selectivity for both metal and oxidation state. By leveraging the activity-based sensing mechanism of metal-directed acyl imidazole chemistry, where probe activation occurs through a tandem sensing and labeling strategy with concomitant release of the metal analyte, we overcome the inherent challenges of turn-on detection with Cu(II), a paramagnetic metal ion that is a potent fluorescence quencher. We anticipate that this strategy will be broadly applicable to other metal ions and related biological analytes that can hinder fluorescence responses. Further tuning of the receptor should afford a generalizable platform for detection of other metals, and increasing the fluorogenicity of the probe upon protein labeling offers a robust strategy for improving signal-to-noise responses (74, 75).

**Fig. 5.**
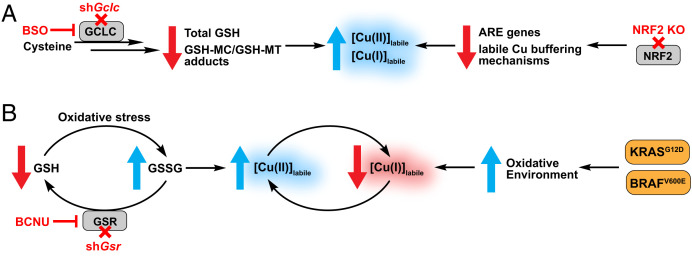
(*A*) Proposed model of how antioxidant metabolism pathways affect the bioavailability of labile copper pools that cycle between Cu(I) and Cu(II) oxidation states. Loss of GSH or NRF2 can cause oxidative stress and lead to aberrant increases in both labile intracellular Cu(I) and Cu(II) stores. (*B*) Proposed model of reciprocal, oxidation state-specific changes in labile intracellular copper pools driven by decreasing GSH/GSSG ratios or expression of oncogenes. Introduction of KRAS or BRAF mutations promote a heightened oxidative environment, resulting in increases in labile Cu(II) pools with corresponding decreases in labile Cu(I) pools.

The unique capabilities of these CDAI reagents have enabled us to study foundational aspects of Cu(II) biology that had previously not been possible. Green-fluorescent **CD517.2** and red-fluorescent **CD649.2** show a Cu(II)-selective response with purified protein and in cell lysates and establish the existence of a labile intracellular Cu(II) pool that can respond to copper supplementation and chelation. Moreover, the combined use of Cu(II)-selective **CD649.2** and a Cu(I)-selective **CF4** probe identify DMT1 and CTR1 as a complementary pair of proteins involved in selective transport of Cu(II) and Cu(I), respectively. Furthermore, we leveraged the Cu(II) specificity of **CD649.2** to investigate the relationship between labile Cu(II) pools and GSH, a major contributor to redox regulation in the cell. We observed that labile Cu(II) levels increase upon pharmacological inhibition or genetic depletion of key regulators in GSH biosynthesis and reduction. These results complement our previous work that linked labile Cu(I) to GSH metabolism (27) and support a model where increased oxidative stress alters the GSH/GSSG ratio and increases the bioavailability of both labile Cu(I) and Cu(II) (Fig. 5). Interestingly, while GSSG can potentially bind to Cu(II) via the carboxyl and amine groups on the glutamate side chain (76), our data show that a decrease in the GSH/GSSG ratio leads to an increase, not a decrease, in labile Cu(II) levels, suggesting a weak affinity of GSSG for Cu(II). Genetic manipulation of *xCT* did not result in significant changes in labile copper or intracellular GSH status; however, it is possible that *xCT* loss was compensated for by additional cysteine transport mechanisms (77), and the effects of perturbing multiple cysteine uptake pathways on labile copper should be further investigated. Additionally, KRAS-transformed cells are known to transcriptionally up-regulate *xCT*, resulting in enhanced GSH levels and protection from oxidative stress (64); indeed, exploring how *xCT* and other GSH regulatory genes might affect labile copper in an oncogenic model could provide more valuable insight into the roles of these genes on metal oxidation state. Loss of NRF2 and the associated antioxidant response leads to an increase in both labile Cu(I) and Cu(II) levels, indicating that the broader antioxidant response to oxidative stress will buffer both forms of the metal. Deciphering antioxidant genes that contribute to sequestering labile copper pools in both oxidation states will yield valuable insights into the emerging relationships between cellular copper and redox status. In this context, metallochaperones like Atox1 that can bind and transport Cu(I) (78), yet also be oxidized by Cu(II) (79), await exploration with the use of Cu(II)-selective probes. Finally, we used **CD649.2** to reveal reciprocal, oxidation state-selective changes in labile intracellular copper pools in a disease-relevant context, showing that expression of oncogenes BRAF^V600E^ or KRAS^G12D^ in MEF cells increases pools of labile intracellular Cu(II) with concomitant decreases in labile intracellular Cu(I) stores (Fig. 5). Many cancer types are associated with aberrantly elevated levels of copper in patient serum (4, 80), where this metal nutrient regulates several proteins involved in tumor growth and proliferation (4, 81). Our findings show that changes in copper oxidation state are also important to consider in these pathways. More broadly, this work provides a starting point for further investigations of metalloplasias, where the metal nutrient pools contribute to metal-dependent disease vulnerabilities by spatial, temporal, and redox control, going beyond traditional metal signals that participate in only one oxidation state.

## Materials and Methods

Full materials and procedures for the synthesis of compounds, spectroscopic characterization, cellular imaging, generation of cell lines, and cell analysis are described in the *SI Appendix*. Reaction monitoring was carried out with a Waters ACQUITY UPLC I-Class PLUS System with PDA Detector and SQ Detector 2 (column: C18, 130 Å, 1.7 µm, 2.1 mm × 30 mm; method: 0–6 min 30% MeCN/70% H_2_O/0.1% TFA (trifluoroacetic acid), flow rate 0.500 mL/min). Metal ion selectivity studies were prepared inside an oxygen-free glovebox, and the results were analyzed by ImageLab. Protein mass spectral analyses were carried out with a Shimadzu Biotech Axima Performance (tuning mode: linear, mass range: 19.0–25.0 kDa, pulsed extraction optimized at 20.6 kDa, matrix: 10 mg/mL sinapinic acid in 50% MeCN/50% H_2_O/0.1% TFA). Cells were imaged with a Zeiss LSM880 laser scanning confocal microscopy system with a 20× dry objective lens, and images were analyzed in Fiji (NIH).

## Supplementary Material

Supplementary File

## Data Availability

All study data are included in the article and/or supporting information.
