# Supplementary material for: Oxidation state-specific fluorescent copper sensors reveal oncogene-driven redox changes that regulate labile copper(II) pools
Source: Proc Natl Acad Sci U S A. 2022 Oct 17;119(43):e2202736119. doi: 10.1073/pnas.2202736119 (PMC9621372; doi:10.1073/pnas.2202736119)
Supplement: Supplementary File [file pnas.2202736119.sapp.pdf]

Supplementary Information for

Oxidation state-specific fluorescent copper sensors reveal oncogene-driven redox changes that regulate labile copper(II) pools

Aidan T. Pezacki[a,1], Carson D. Matier[a,1], Xingxing Gu[d], Eric Kummelstedt[a], Sarah E. Bond[e], Laura Torrente[f], Kelly L. Jordan-Sciutto[e], Gina M. DeNicola[f], Timothy A. Su[a,g,h]*, Donita C. Brady[d,i]*, and Christopher J. Chang[a,b,c]*

[a]Department of Chemistry, [b]Department of Molecular and Cell Biology, and [c]Helen Wills Neuroscience Institute, University of California, Berkeley, California 94720, United States; [d]Department of Cancer Biology, Perelman School of Medicine, University of Pennsylvania, Philadelphia, PA 19104, United States; [e]Department of Oral Medicine, School of Dental Medicine, University of Pennsylvania, Philadelphia, PA 19104, United States; [f]Department of Cancer Physiology, H. Lee Moffitt Cancer Center and Research Institute, Tampa, FL 33612, United States; [g]Department of Chemistry, University of California, Riverside, CA 92521, United States; [h]Materials Science and Engineering Program, University of California, Riverside, CA 92521; [i]Abramson Family Cancer Research Institute, Perelman School of Medicine, University of Pennsylvania, Philadelphia, PA 19104, United States

[1]These authors contributed equally

[2]Corresponding Authors: *E-mail: chrischang@berkeley.edu; bradyd@pennmedicine.upenn.edu; timothys@ucr.edu

This PDF includes:

Supplementary Text

Figures S1 to S24

Synthetic Protocols and Characterization Data

SI References

General Methods. Reagents were purchased from commercial sources and used without further purification unless otherwise noted. Standard Schlenk line techniques with N_2 atmosphere were utilized for synthetic manipulations unless otherwise noted. Anhydrous dichloromethane (CH_2Cl_2), dimethylformamide (DMF), and tetrahydrofuran (THF) were dried and purified using a solvent purification system (JC Meyer) under an argon atmosphere. Thin-layer chromatography (TLC) analysis of reaction mixtures were performed using Merck silica gel 60 F254 TLC plates and visualized using KMnO4 stain or UV. Column chromatography was carried out using Merck silica gel (60 Å, 230 X 400 mesh). ^{1}H, ^{13}C, and ^{19}F NMR experiments were conducted using either Bruker 300, 500, or 600 MHz NMR instruments in deuterated solvent (Cambridge Isotope Laboratories, Cambridge, MA). Peaks were referenced using residual solvent peaks and chemical shifts are reported in δ parts per million (ppm). Splitting patterns are indicated as follows: br, broad; s, singlet; d, doublet; t, triplet; m, multiplet; dd, doublet of doublets. Low-resolution electrospray mass spectral analyses were carried out using a Advion expression-L Compact Mass Spectrometer with direct injection on positive ion mode. High-resolution mass spectral analyses (HR-ESI-MS) were carried out at the College of Chemistry Mass Spectrometry Facility at the University of California, Berkeley. Reaction trace monitoring was carried out using a Waters ACQUITY UPLC I-Class PLUS System with PDA Detector and SQ Detector 2 (column: C18, 130Å, 1.7 µm, 2.1 mm X 30 mm, method: 0 to 6 minutes 30% MeCN/70% H2O/0.1% TFA, flow rate 0.500 mL/min). Protein mass spectral analyses were carried out using a Shimadzu Biotech Axima Performance (tuning mode: linear, mass range: 19.0-25.0 kDa, pulsed extraction optimized at 20.6 kDa, matrix: 10 mg/mL sinapinic acid in 50% MeCN/50% H2O/0.1% TFA). Absorbance measurements were carried out using an Agilent Technologies Cary 60 UV-Vis in Sarstedt AG & Co. KG 10 x 4 x 45 mm polystyrene cuvettes.

***In Vitro* Protein Labeling.** Metal ion selectivity studies were prepared inside an oxygen-free glovebox. For metal ion selectivity studies using a model protein, various metal ions (1 mM NaCl, 1 mM KCl, 1 mM $CaCl_2$, 1 mM $MgCl_2$, 1 mM $MnCl_2$, 10 µM $FeSO_4$, 10 µM $CoSO_4$, 10 µM Vitamin B_{12}, 10 µM CuCl, 10 µM $CuSO_4$, 10 µM $CuSO_4$ with 50 µM BCS or 10 µM $ZnSO_4$) were added to 1.55 µM soybean trypsin inhibitor (SBTI, Sigma-Aldrich) in PBS and equilibrated for 5 minutes at room temperature. 10 µM **CD649.2** (dissolved in DMSO) was added and incubated with the protein and metal ions for 30 minutes at room temperature. The final volume was 50 µL. The solution mixture was mixed with NuPAGE® LDS Sample Buffer (4X) and heated at 95 ^{o}C for 5 minutes, followed by separation on Novex tris-glycine gels (Invitrogen) and scanned by ChemiDoc MP (Bio-Rad Laboratories, Inc) for measuring in-gel fluorescence. The fluorescence of **CD649.2** was measured by 695/55 nm band pass filter with excitation using red epi-illumination. After that, the total protein level on the gel was assayed by silver staining (ThermoFisher Scientific; 24612) according to the manufacturer's protocol and scanned by ChemiDoc MP. Metal ion selectivity studies using cell lysate was similarly prepared. To prepare cell lysates, cultured HEK 293T cells were rinsed with PBS, suspended in lysis buffer (100 mM HEPES, 58.5 mM NaCl, pH 7.4), homogenized by ultrasonic treatment, and centrifuged (2,740 G at 4 °C for 7 minutes). The supernatant was collected, and the protein concentrations were measured by BCA Protein Assay Kit (Thermo-Fisher). HEK 293T cell lysate (0.2 mg/mL) were preincubated with biologically relevant metal ions for 5 minutes (final conc. of *s*-block metal ion = 1 mM; final conc of *d*-block metal ion = 30 µM; BCS = 150 µM). Subsequently, 30 µM **CD649.2** (dissolved in DMSO) was added and the mixture was incubated at room temperature for 30 minutes. The final volume was

25 µL. The protein mixture was then separated by SDS-PAGE and imaged by ChemiDoc MP as above. The integrated intensities were analyzed by ImageLab.

Cell Culture. Cells were maintained by the UC Berkeley Tissue Culture Facility. HEK 293T cells were maintained as a monolayer in exponential growth at 37 °C in a 5% CO_2 atmosphere in Dulbecco's Modified Eagle Medium (DMEM, Gibco) supplemented with 10% fetal bovine serum (FBS, Seradigm), 1% glutamax (Gibco), and 1% non-essential amino acids (NEAA, Gibco). H1299 Ctr1 KO, DMT1 KO, Double KO, and WT cells were obtained from Massimo Broggini (Istituto di Ricerche Farmacologiche Mario Negri) and were maintained as a monolayer in exponential growth at 37 °C in a 5% CO_2 atmosphere in Roswell Park Memorial Institute 1640 Medium (RPMI 1640, Corning) supplemented with 10% fetal bovine serum (FBS, Seradigm), and L-glutamine (Corning). Two days before imaging, cells were passed and plated on 8-well chamber slide coated with poly L-lysine (50 mg/mL, Sigma, St. Louis, MO).

MEF cells were immortalized with a plasmid expressing SV40 gene. Immortalized MEFs were cultured in Dulbecco's Modified Eagle Medium (DMEM, Gibco) supplemented with 10% v/v fetal bovine serum (FBS, Hyclone Laboratories), 1% v/v Penicillin/ Streptomycin (P/S, Gibco) as described previously. MEFs stably expressing shScr, sh*Gclc*, sh*Gsr*, or $BRAF^{V600E}$, $KRAS^{G12D}$ were maintained in DMEM supplemented with 10% FBS, 1% P/S, and 2 µg/mL puromycin or 5 µg/mL blasticidin (Invitrogen). $NRF2^{-/-+V0}$ and $NRF2^{-/-+NRF2}$ A549 cell lines were obtained from Gina DeNicola (Moffitt Cancer Center) and were maintained in DMEM supplemented with 10% FBS, 1% P/S, and 2 µg/mL puromycin.

Cell Viability Assay. Hoechst 33342 was used to confirm cell viability upon treatment with CD649.2 and copper supplementation/chelation. HEK 293T cells were cultured and treated using identical protocols as reported in cell culture and confocal fluorescence imaging, with the exception of 0.2 µg/mL Hoechst 33342 being co-incubated with **CD649.2**. Fluorescence intensity of Hoechst was determined with λex = 405 nm. Presto Blue (Thermo Fisher) was used to further confirm cell viability. HEK 293T cells were plated at 10,000 cells/well in a 96-well plate in DMEM/10% FBS. Cells were incubated with 100 µL of vehicle control, copper chelate for 24 hours or ionophore for 1 hour in DMEM/10% FBS at 37°C/5% CO_2. Cells were then washed once with DMEM/10% FBS, and washed once with HBSS (+Ca, Mg). The buffer solution was replaced with 100 µL of vehicle control, 1 µM **CD649.2**, or 50-500 µM H_2O_2 in HBSS (+Ca,Mg), and incubated at 37°C/5% CO_2 for 1 hour. The solution was replaced with 90 µL HBSS (+Ca, Mg) + 10 µL Presto Blue and incubated at 37°C/5% CO_2 for 90 minutes. Fluorescence was then read on a plate reader (monofilter) with excitation wavelength at 560 nm (9 mm bandwidth), emission wavelength at 590 nm (9 mm bandwidth), 15 mm plate height and top-down fluorescence detection.

Plasmids. sh*Gclc* and sh*Gsr* MEFs were infected with lentiviruses derived from TRC cloning vector pLKO.1 (TRCN0000112453, TRCN0000112452 for Gclc, TRCN0000042338, TRCN0000042339 for *Gsr*) using established protocols. sh*xCT* MEFs with parental *Ctr1* were infected with lentiviruses derived from TRC cloning vector pLKO.1 (TRCN0000311401, TRCN0000079427) using established protocols. MEFs stably expressing xCT shRNA were selected for using 4 µg/mL puromycin for 1 week, at which point selection was confirmed by parallel death of all non-infected parental Ctr1 MEFs in selection media. All shRNA MEFs were maintained with 2 µg/mL puromycin. $BRAF^{V600E}$ and $KRAS^{G12D}$ MEFs were stably infected with retroviruses derived from pBABEpuro-MYC-HIS-BRAFV600E (Addgene plasmid #15269) or

pBABEpuro-HA-KRASG12D (Addgene plasmid #58902) respectively, using established protocols.

Reverse Transcriptase Quantitative PCR. RNA was extracted from MEF cells and reverse transcribed to cDNA. mRNA levels were quantified with Taqman probes: Mm00439154_m1 to detect mouse Gsr, Mm00802655_m1 to detect mouse Gclc, and Mm00442530_m1 to detect mouse xCT. Relative mRNA expression levels were normalized to Tbp using comparative ΔΔCT method and represented as fold change.

Confocal Fluorescence Imaging. HEK 293T cells and H1299 cells (wildtype, DMT1 KO, and CTR1 KO) were seeded at 20-40% confluency in a poly L-lysine coated 8-well chamber slide (Nunc Lab-Tek) and were allowed to grow to 70% confluency before performing cell imaging. HEK 293T cells were incubated with 1 µM Cu(gtsm) for 1 hour, 400 µM TETA for 24 hours, or 200 µM DPA for 24 hours in DMEM/10% FBS medium with 1% DMSO. Cells were then washed twice with HBSS (+Ca, Mg), followed by incubation with 1 µM **CD649.2** in HBSS (+Ca, Mg) with 2% DMSO for 1 hour. Cells were washed once with HBSS (+Ca, Mg) and imaged. WT, DMT1 KO, and CTR1 KO H1299 cells were incubated with 200, 350, or 500 µM TETA, DPA, or vehicle control in RMPI/10% FBS medium with 1% DMSO for 24 hours. Cells were then washed twice with HBSS (+Ca, Mg), followed by incubation with 1 µM **CD649.2** in HBSS (+Ca, Mg) with 2% DMSO for 30 minutes. Cells were washed once with HBSS (+Ca, Mg) and imaged. Confocal fluorescence imaging with HEK 239T cells and H1299 cells with DMT1 KO, CTR1 KO was performed with a Zeiss laser scanning microscope LSM880 with a 20x dry objective lens using Zen 2015 software (Carl Zeiss, Zen 2.3 black). CD649.2 was excited using a 633 nm diode laser and emission was collected using a META detector between 650 to 750 nm. Z-slices were taken from the top to the bottom of the cells for each image.

For confocal microscopy imaging experiments with MEFs stably expressing with shScr, sh*Gclc*, sh*Gsr*, or $BRAF^{V600E}$, $KRAS^{G12D}$ and $NRF2^{-/-+V0}$, $NRF2^{-/-+NRF2}$ A549 cells, cells were seeded at 250,000 cells/plate in 35 mm glass insert imaging plates (MatTek) and 48 hours later were imaged. MEFs stably expressing sh*xCT* and parental lines were seeded at 100,000 cells/plate in 35 mm glass insert imaging plates (Greiner BioOne) and 48 hours later were imaged. Cells were washed twice with HBSS (1X,+ Ca, Mg, w/o Phenol Red, Corning), followed by incubation with 1 µM **CD649.2** in HBSS solution for 1 hour. For BSO or BCNU treatment, cells were treated with vehicle, 1 mM BSO, or 0.1 mM BCNU in complete medium for 4 hours followed by incubation of 1 µM **CD649.2** in HBSS. Cells were imaged on a Zeiss LSM880 laser scanning confocal microscopy system with a 20x dry objective lens. **CD649.2** was excited by a 633 nm HeNe laser, and emissions were collected between 640 nm and 758 nm.

Image Analysis and Quantification. Fiji (National Institutes of Health) was used for image analysis. The area of stained cells was selected by setting the appropriate threshold for the z-projection (max intensity) of the Z-slices with a Gaussian blur filter (sigma = 1). The "Create Mask" function followed by the "Create Selection" function were then used to create a selection from this threshold. Using this selection, the mean fluorescence of each Z-slice was measured by the "Multi-Measure" function. The average fluorescence intensity of each set was obtained by taking the average of the maximum fluorescence intensity Z-slice plus the two Z-slices directly above or below the maximum fluorescence slice. For each condition, four sets of Z slices in different fields of cells were analyzed from each biological replicate using this process and the values were combined for statistical analysis. Statistical comparison of **CD649.2** fluorescence in different

conditions versus control was performed using an unpaired t-test (assume Gaussian distribution, no Welch's correction) in Prism9 (GraphPad).

Microscope images of MEFs stably expressing with shScr, sh*Gclc*, sh*Gsr*, sh*xCT*, or *BRAF*^{V600E}, *KRAS*^{G12D} and *NRF2*$^{-/-+V0}$, *NRF2*$^{-/-+NRF2}$ A549 cells were quantified using Image J software. For quantification, fluorescence intensity of **CD649.2** was determined from experiments performed in triplicate in which 10 single cells per field of view from three independent images for a total of 90 cells analyzed across three biological replicates. The mean intensity was used to do final statistical analysis. Statistical analysis of normalized **CD649.2** fluorescence per cell was performed using a one-way ANOVA followed by a Tukey's multi-comparisons test in Prism7 (GraphPad).

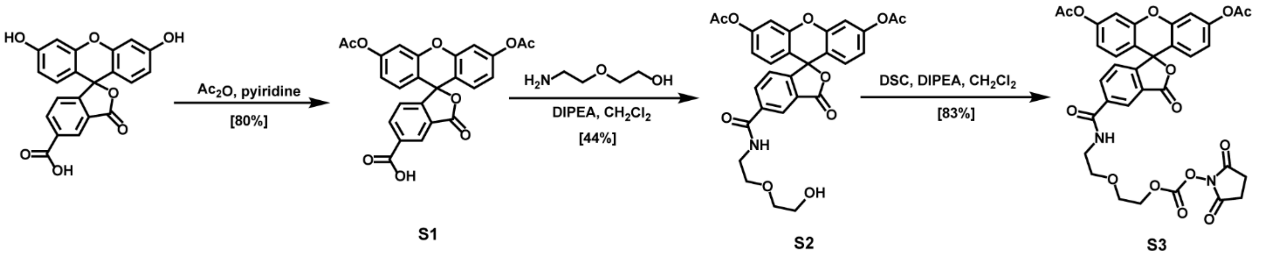

Scheme S1. Synthesis of **CD517.2**, a green-fluorescent Cu(II)-selective Copper-Directed Acyl Imidazole probe bearing a fluorescein dye reporter.

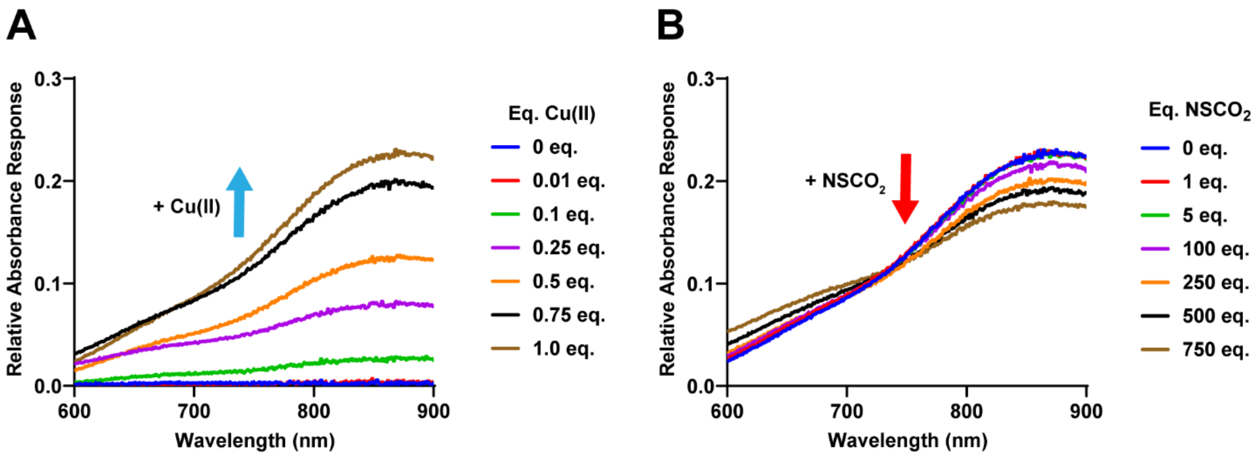

Fig. S1. Ligand competition assay reveals the Cu(II) binding affinity of **$NSCO_2$**. (A) Absorbance spectra of Tris(2-pyridylmethyl)amine (TPA) with varying equivalence of Cu(II). 1 mM TPA was incubated with 0-1 equivalence of $CuCl_2$ in H_2O at room temperature for 15 minutes. An absorbance peak at 880 nm increases in intensity for increasing Cu(II) concentration. (B) Absorbance spectra of Cu(II)-bound TPA with varying equivalence of **$NSCO_2$**. 1 mM TPA was incubated with 1 mM $CuCl_2$ and 0-750 equivalence of **$NSCO_2$** in H_2O at room temperature for 15 minutes. As the concentration of **$NSCO_2$** increases, the absorbance of the Cu(II)-TPA complex decreased, suggesting displacement of bound Cu(II) from TPA to $NSCO_2$ and hence supporting the strong binding affinity for Cu(II) of $NSCO_2$. From the following data, a K_d of 1.93×10^{-14} M was calculated using previously reported methods (1).

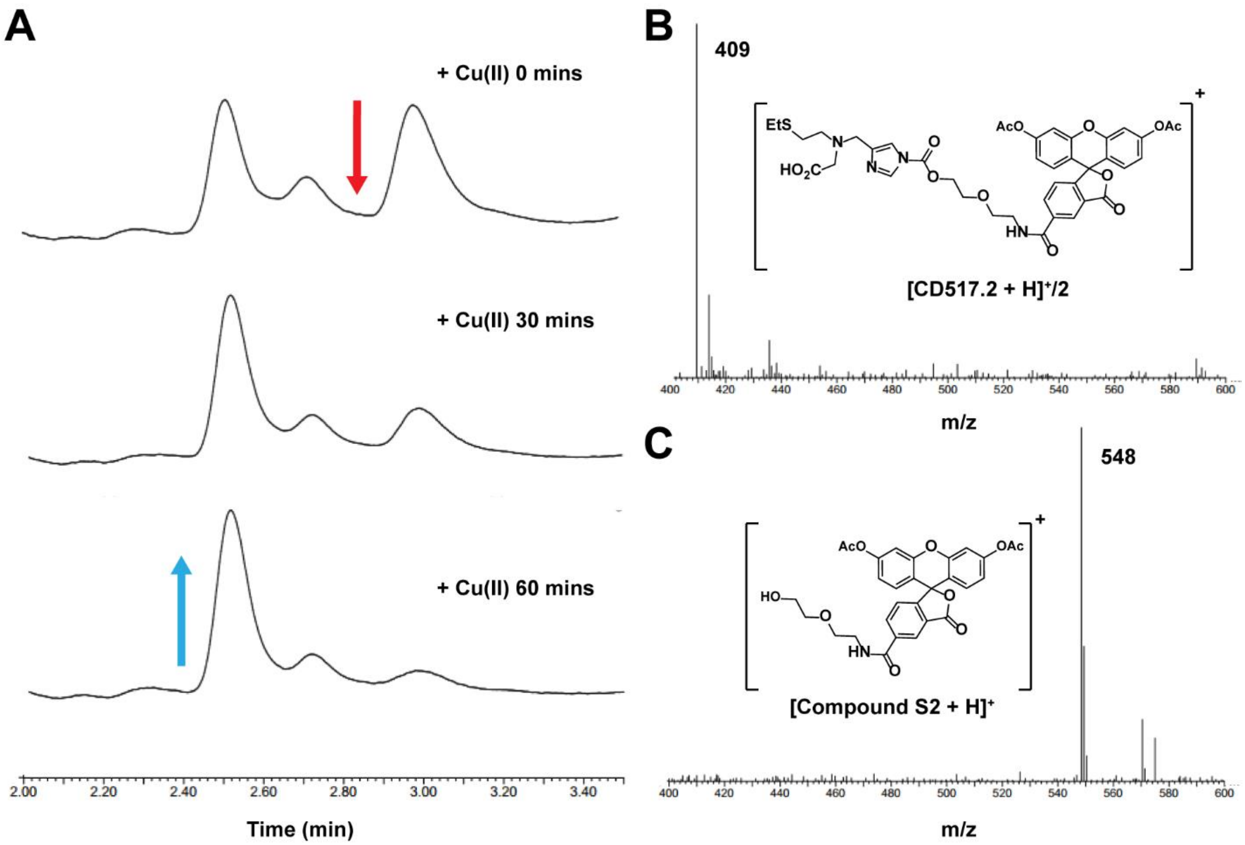

Fig. S2. Reactivity of **CD517.2** and Cu(II) in aqueous buffer solution. (A) LC chromatograms of the reaction between 100 μM **CD517.2** and 250 μM $CuSO_4$ in PBS for 1 h, showing near complete conversion of **CD517.2** to compound **1**. (B) The mass of intact **CD517.2** was detected at a retention time of 3.2 minutes. (C) The hydrolyzed product of **CD517.2** and Cu(II) is detected at a retention time of 2.5 minutes.

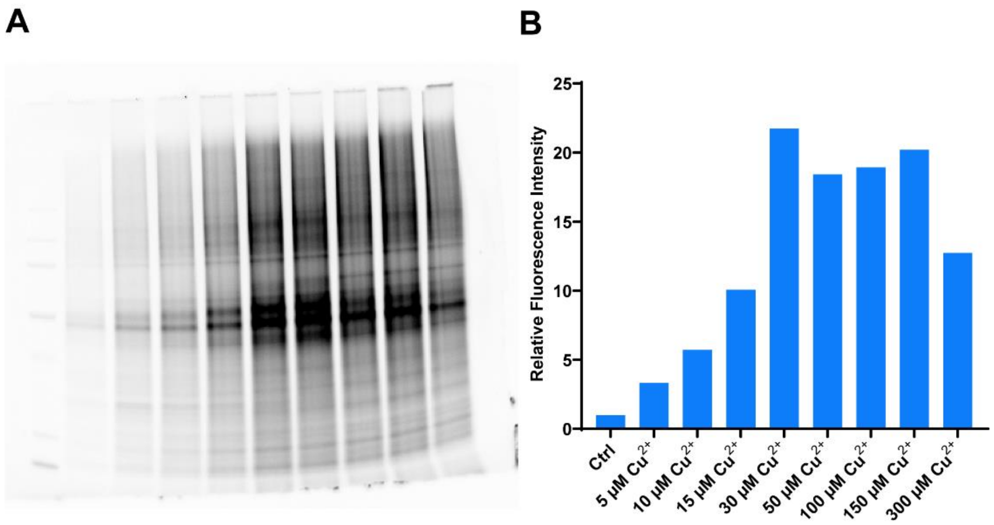

Fig. S3. CD649.2 can detect Cu(II) in a dose-dependent manner by proximal protein labeling using metal-directed acyl imidazole chemistry. (A) In-gel fluorescence image and (B) integrated fluorescence intensities of HEK 293T cell lysates treated with **CD649.2** and Cu(II). Cell lysate (0.2 mg/mL) was preincubated with varying concentrations of $CuCl_2$ for 5 minutes, followed by incubation with 30 μM **CD649.2** at room temperature for 30 minutes. In-gel fluorescence for SDS-PAGE was scanned by ChemiDoc MP and signal intensity was analyzed by Image Lab.

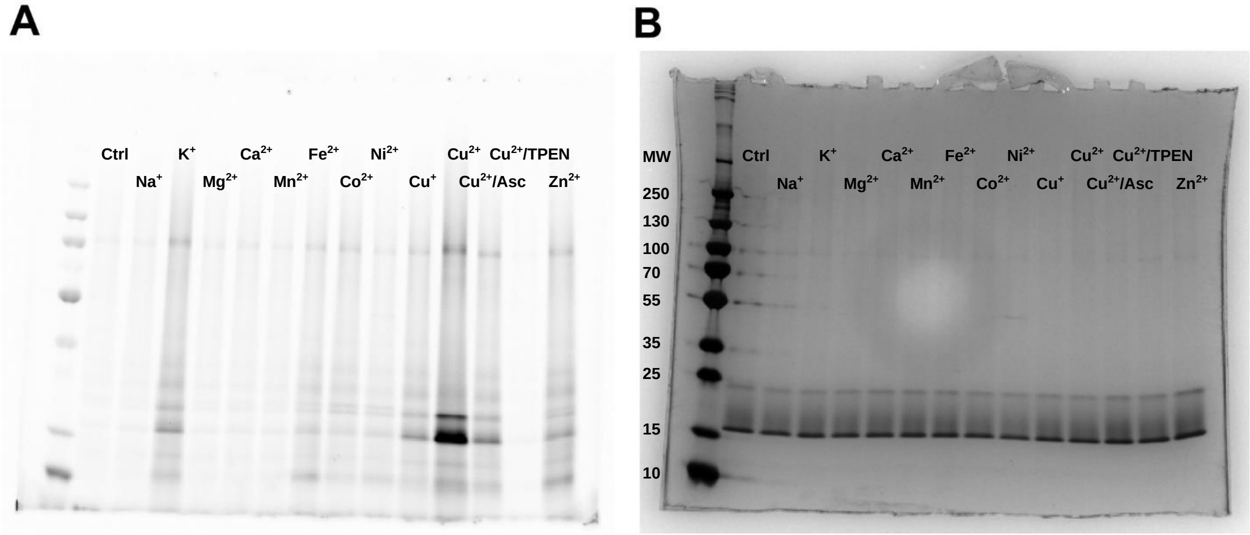

Fig. S4. Representative full images of SDS–PAGE analysis on soybean trypsin inhibitor (SBTI) treated with various biologically relevant metals and **CD649.2**. (A) Protein labeling by **CD649.2**, as measured by in-gel fluorescence. (B) SBTI concentration, as measured by Pierce™ Silver Stain Kit.

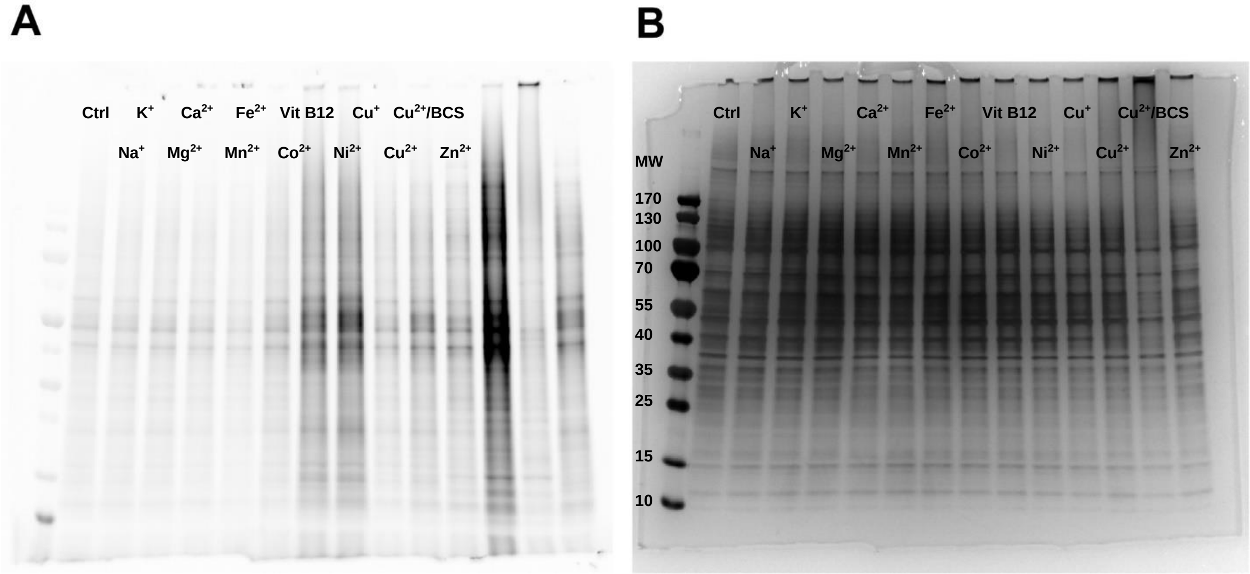

Fig. S5. Representative full images of SDS–PAGE analysis on HEK 293T cell lysates treated with various biologically relevant metals and **CD649.2**. (A) Protein labeling by **CD649.2**, as measured by in-gel fluorescence. (B) HEK 293T cell lysate protein concentration, as measured by Pierce™ Silver Stain Kit.

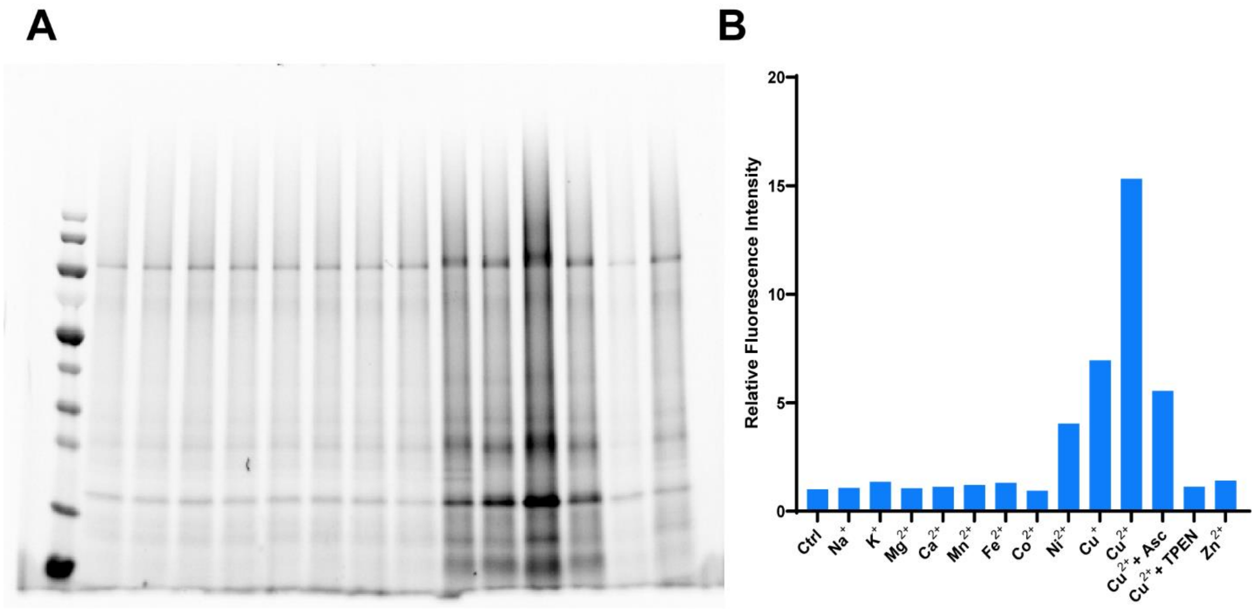

Fig. S6. The first-generation **CD649** reagent shows only modest oxidation state selectivity for Cu(II) over Cu(I) in protein labeling assays. (A) In-gel fluorescence image and (B) integrated fluorescence intensities of **CD649** and soybean trypsin inhibitor (SBTI) as a model protein. SBTI (10 µM) was preincubated with biologically relevant metal ions for 5 minutes (s-block metal ions at 1 mM, d-block metal ions at 10 µM, TPEN at 50 µM, Ascorbate at 30 µM), followed by incubation with 10 µM **CD649** at room temperature for 30 minutes. In-gel fluorescence for SDS-PAGE was scanned by ChemiDoc MP and signal intensity was analyzed by Image Lab.

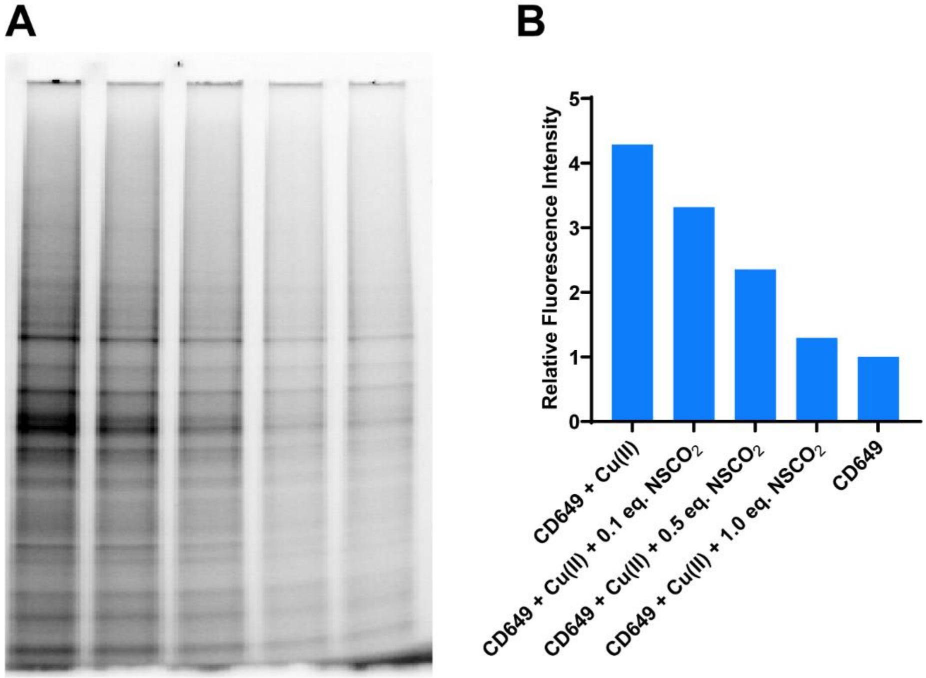

Fig. S7. NSCO₂ can competitively chelate Cu(II) and prevent proximal protein labeling by CD649 via metal-directed acyl imidazole chemistry. (A) In-gel fluorescence image and (B) integrated fluorescence intensities of HEK 293T cell lysates treated with **CD649**, 10 μM Cu(II), and varying equivalence of **$NSCO_2$**. Cell lysate (0.2 mg/mL) was preincubated with 10 μM $CuCl_2$ or control for 5 minutes, followed by incubation with 10 μM **CD649** and 1-10 μM **$NSCO_2$** at room temperature for 30 minutes. In-gel fluorescence for SDS-PAGE was scanned by ChemiDoc MP and signal intensity was analyzed by Image Lab.

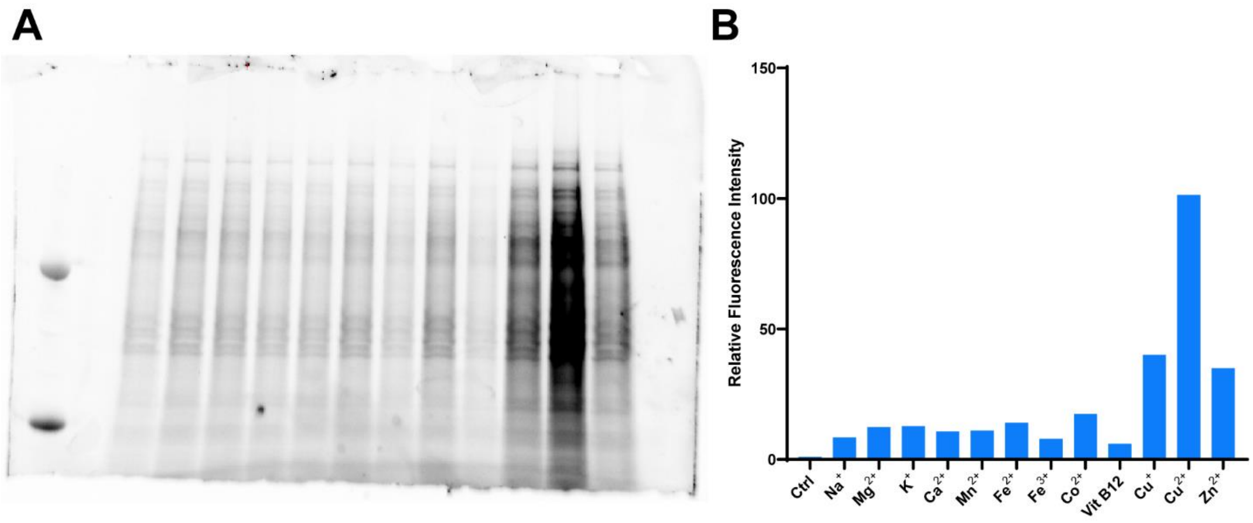

Fig. S8. CD517.2 shows metal and oxidation state selectivity for Cu(II) compared to other biologically relevant metal ions in the presence of HEK 293T cell lysates. (A) In-gel fluorescence image and (B) integrated fluorescence intensities of **CD517.2** and HEK 293T cell lysates. Cell lysate (0.5 mg/mL) was preincubated with biologically relevant metal ions for 5 minutes (s-block metal ions at 100 mM, d-block metal ions at 500 μM, BCS at 50 μM), followed by incubation with 50 μM **CD517.2** at room temperature for 30 minutes. In-gel fluorescence for SDS-PAGE was scanned by ChemiDoc MP and signal intensity was analyzed by Image Lab.

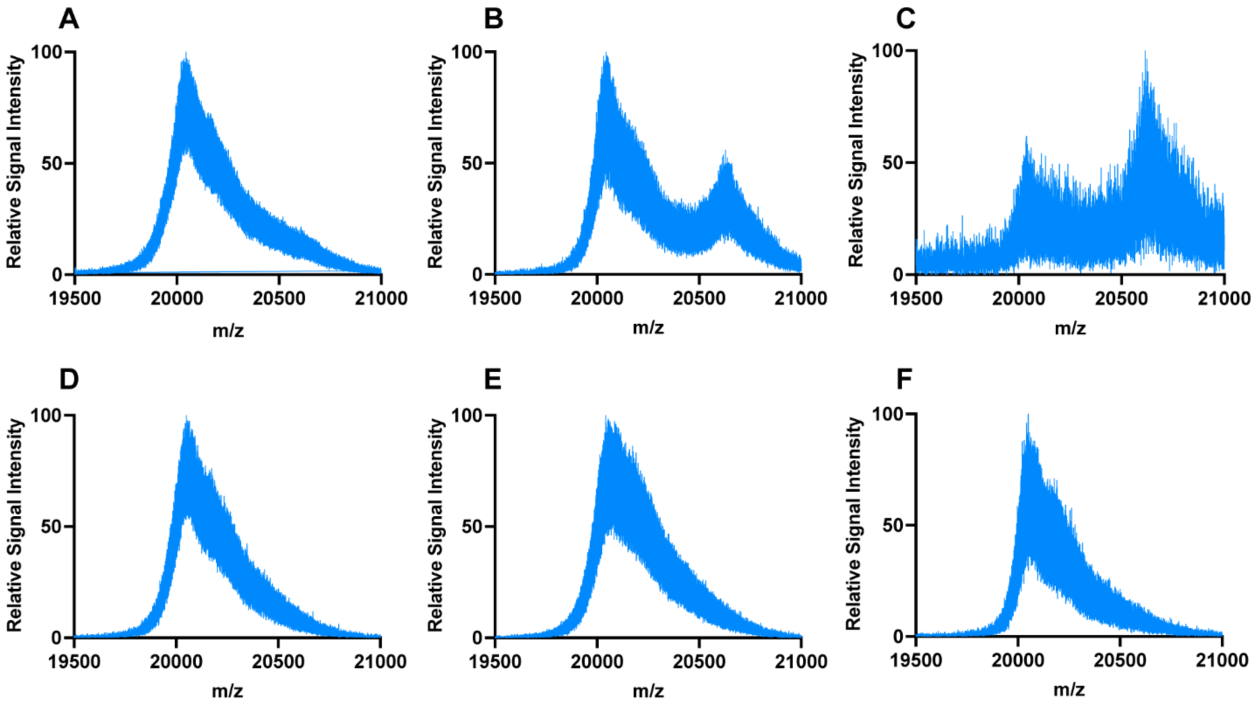

Fig S9. CD649.2 labels SBTI in a Cu(II)-dependent manner. Mass spectra of SBTI treated with **CD649.2** and 50 μM (A), 100 μM (B), or 250 μM (C) Cu(II); 50 μM (D), 100 μM (E), or 250 μM (F) Cu(I). SBTI (5 mg/mL) was preincubated with biologically relevant metal ions for 5 minutes followed by incubation with 50 μM **CD649.2** at room temperature for 30 minutes. The mass adduct of SBTI (20.1 kDa) + conjugated probe (603.75 Da) was detected only for 100 and 250 μM Cu(II) demonstrating enhanced Cu(II)-selectivity for metal-induced protein labeling by **CD649.2**. Spectra were obtained via matrix-assisted laser desorption/ionization time-of-flight (MALDI-TOF) mass spectrometry (matrix: 10 mg/mL sinapinic acid in 50% MeCN/50% H2O/0.1% TFA).

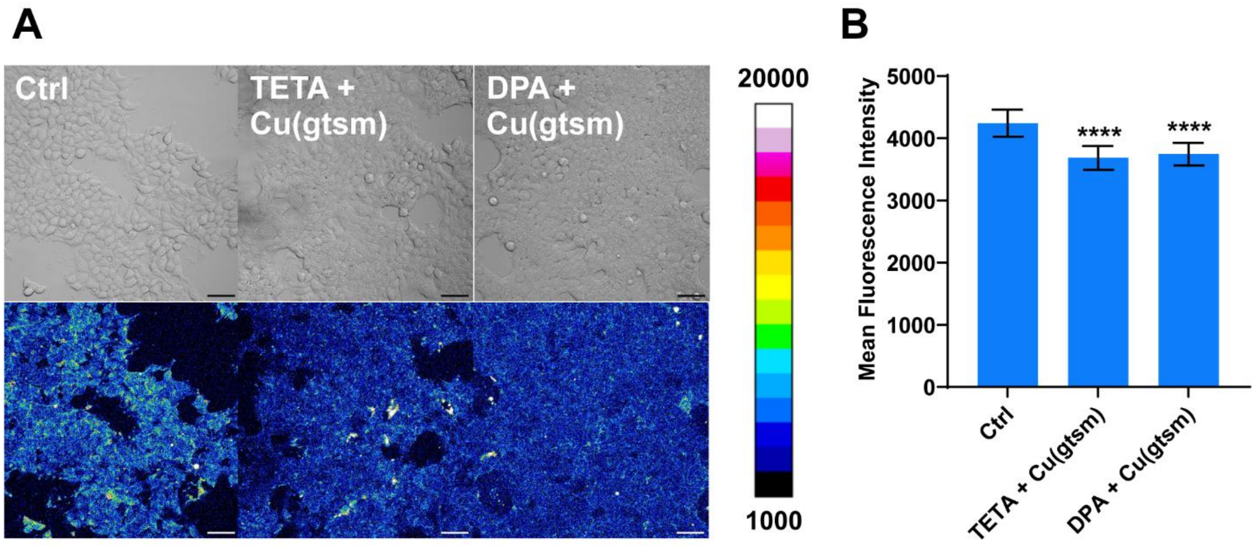

Fig. S10. CD649.2 is capable of detecting exogenous labile Cu(II) chelation. (A) Confocal fluorescence microscopy images of HEK 293T cells treated with solvent vehicle control, 200 µM TETA and 2 µM Cu(gtsm), or 200 µM DPA and 2 µM Cu(gtsm). Cells were incubated with chelator or vehicle control in DMEM/10% FBS for 24 hours, washed once with DMEM, and treated with Cu(gtsm) or vehicle control for 1 hour in DMEM. Cells were then washed twice with HBSS, incubated with 1 µM **CD649.2** in HBSS for 1 hour, washed once with HBSS, and then imaged. (B) Normalized cellular fluorescence intensities of the HEK 293T cells as determined using ImageJ, showing a decrease in fluorescence signal when treated with TETA/DPA followed by Cu(gtsm). Fluorescence intensity of **CD649.2** was determined from experiments performed in triplicate with $\lambda ex = 633$ nm. Error bars denote standard deviation (SD; n = 12). Scale bar = 50 µm. $*p < 0.05$, $**p < 0.01$, $***p < 0.001$, and $****p < 0.0001$; ns, not statistically significant.

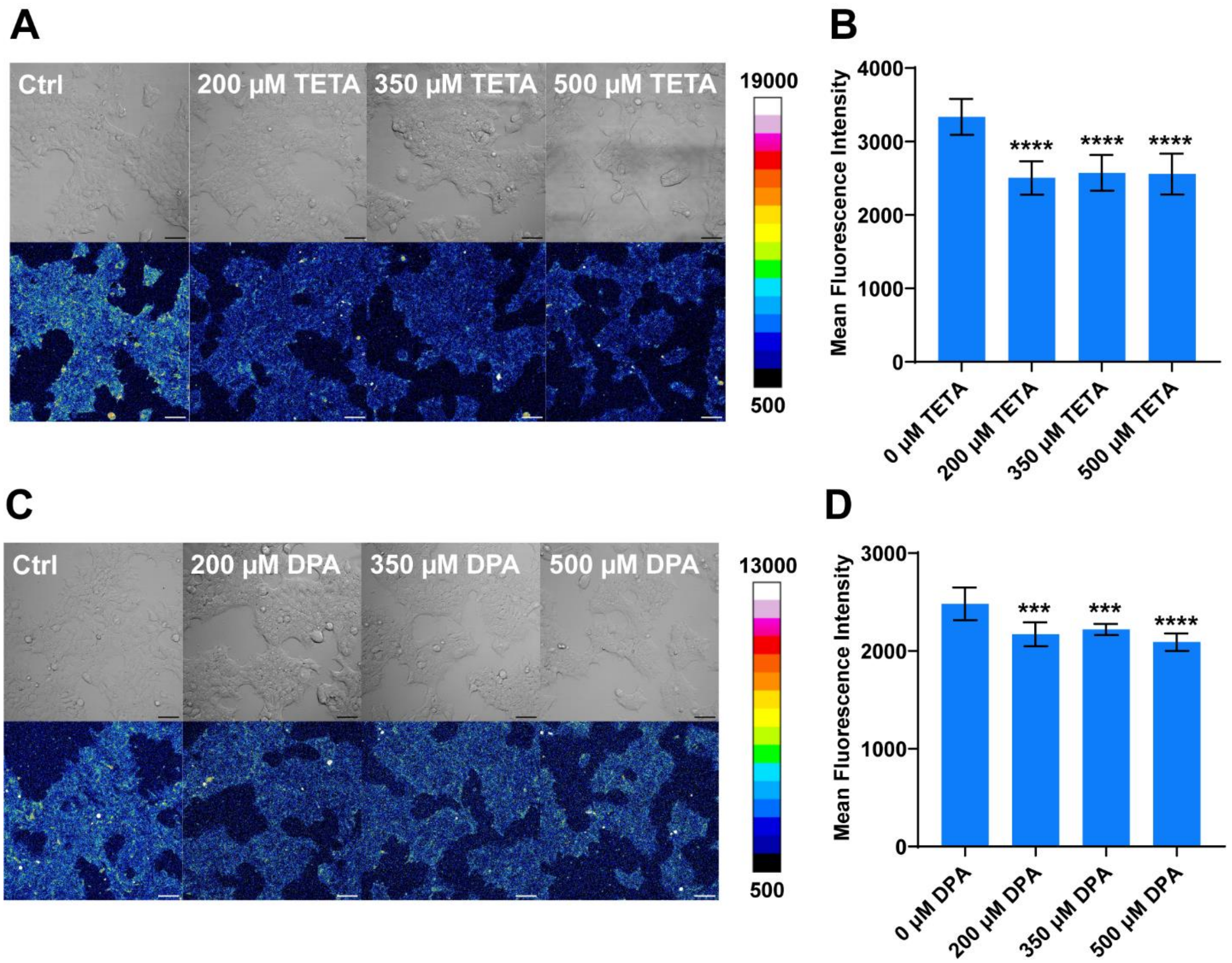

Fig. S11. CD649.2 can detect decreases in endogenous labile Cu(II) in HEK 293T cells in response to treatment with TETA or DPA. Confocal fluorescence microscopy images of HEK 293T cells incubated with 200-500 µM TETA (A), 200-500 µM DPA (C), or vehicle control in DMEM/10% FBS medium with 1% DMSO for 24 hours. Cells were then washed twice with HBSS (+Ca, Mg), followed by incubation with 1 µM **CD649.2** in HBSS (+Ca, Mg) with 2% DMSO for 1 hour. Cells were washed once with HBSS (+Ca, Mg) and imaged. Normalized cellular fluorescence intensities of the HEK 293T cells as determined using ImageJ, showing no dose-dependent decrease in fluorescence signal in response to increasing concentration of TETA (B) or DPA (D). Fluorescence intensity of **CD649.2** was determined from experiments performed in triplicate with λex = 633 nm. Error bars denote standard deviation (SD; n = 12). Scale bar = 50 µm. *$p < 0.05$, **$p < 0.01$, ***$p < 0.001$, and ****$p < 0.0001$; ns, not statistically significant.

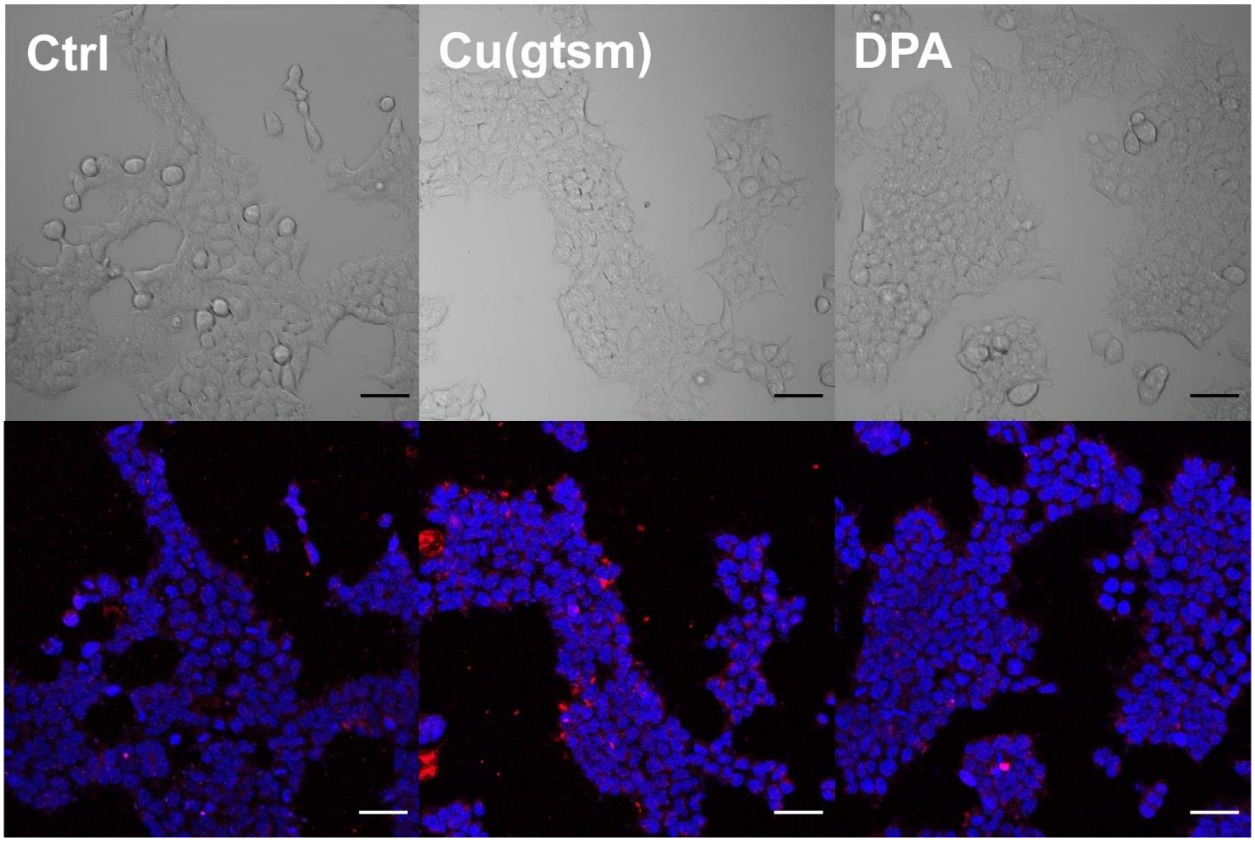

Fig. S12. Dual-color imaging experiments with Hoechst 33342 and **CD649.2** reveal high cell viability and cytoplasmic and nuclear localization of labile Cu(II) pools. Confocal fluorescence microscopy images of HEK 293T cells treated with solvent vehicle control, 1 µM Cu(gtsm) for 1 hour, or 200 µM DPA in DMEM/10% FBS with 1% DMSO for 24 hours. Cells were washed twice with HBSS (+Ca, Mg), incubated with **CD649.2** and Hoechst 33342 in HBSS (+Ca, Mg) with 2% DMSO for 1 hour, washed once with HBSS, and then imaged. **CD649.2** was excited with λex = 633 nm while Hoechst 33342 was excited with λex = 405 nm.

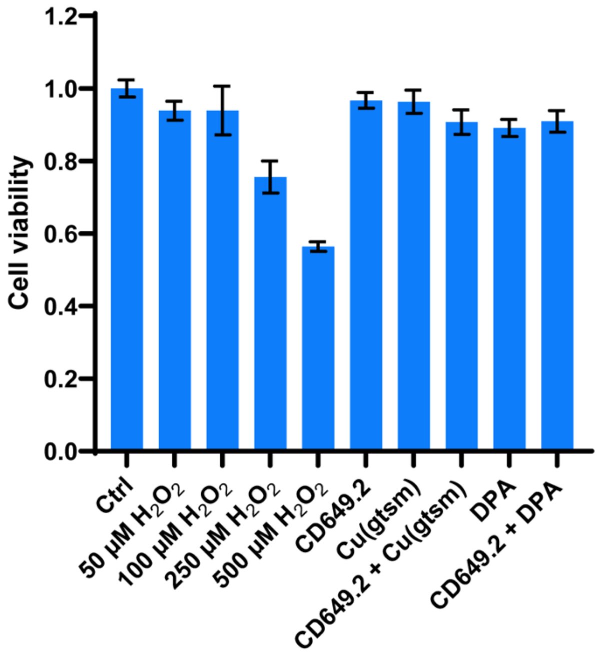

Fig. S13. HEK 293T cell viability remains high in the presence of **CD649.2** and other pharmacological treatments. Cells were incubated with vehicle control, DPA for 24 hours, or ionophore for 1 hour in DMEM/10% FBS at 37°C/5% CO_2, washed once with DMEM/10% FBS, and washed once with HBSS. Cells were incubated with vehicle control, 1 µM **CD649.2**, or 50-500 µM H_2O_2 in HBSS and incubated at 37°C/5% CO_2 for 1 hour. The solution was replaced with 90 µL HBSS + 10 µL Presto Blue and incubated at 37°C/5% CO_2 for 90 minutes. Fluorescence was then read on a plate reader (monofilter) with excitation wavelength at 560 nm (9 mm bandwidth), emission wavelength at 590 nm (9 mm bandwidth), 15 mm plate height and top-down fluorescence detection.

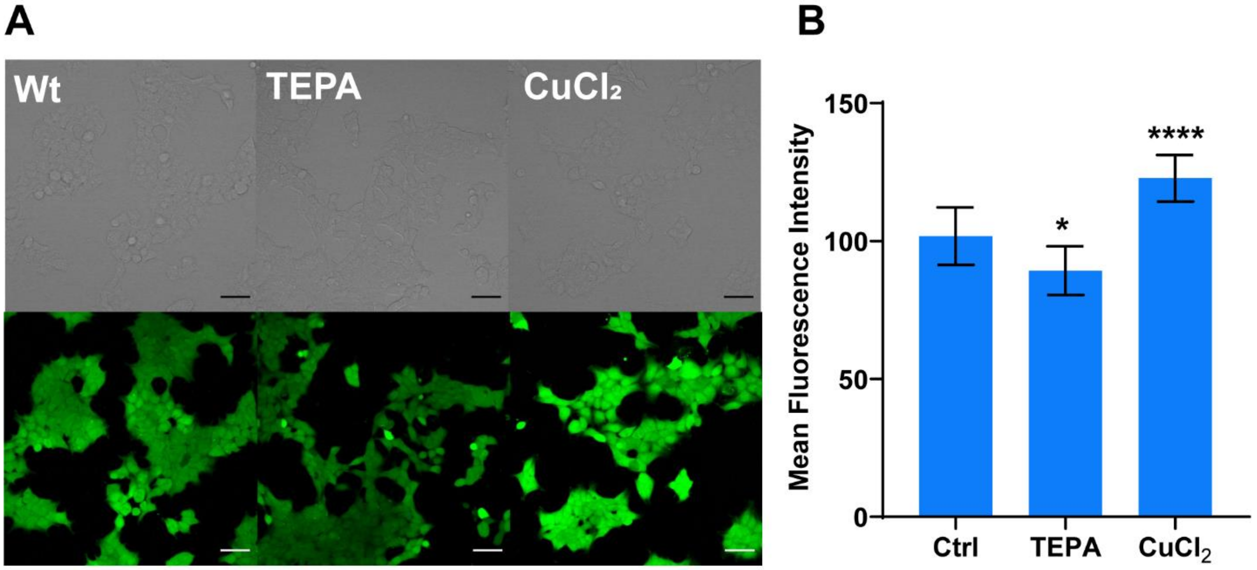

Fig. S14. CD517.2 is capable of detecting changes in intracellular labile Cu(II) pools via live-cell confocal microscopy. (A) Confocal fluorescence microscopy images of HEK 293T cells treated with solvent vehicle control, 100 µM $CuCl_2$, or 50 µM Tetraethylenepentamine (TEPA) in DMEM/10% FBS for 2 hours. The media was then replaced with 2 µM **CD517.2** in DPBS (+Ca, Mg) for 30 minutes, washed once with DPBS (+Ca, Mg), and then imaged. (B) Normalized cellular fluorescence intensities of the HEK 293T cells as determined using ImageJ, showing a turn-on response when treated with $CuCl_2$ and a decrease in fluorescence signal in response to TEPA. **CD517.2** was excited with λex = 488 nm and fluorescence was measured using an emission spectral window from 510 to 650 nm. Error bars denote standard deviation (SD; n = 8). Scale bar = 50 µm. *p < 0.05, **p < 0.01, ***p < 0.001, and ****p < 0.0001; ns, not statistically significant.

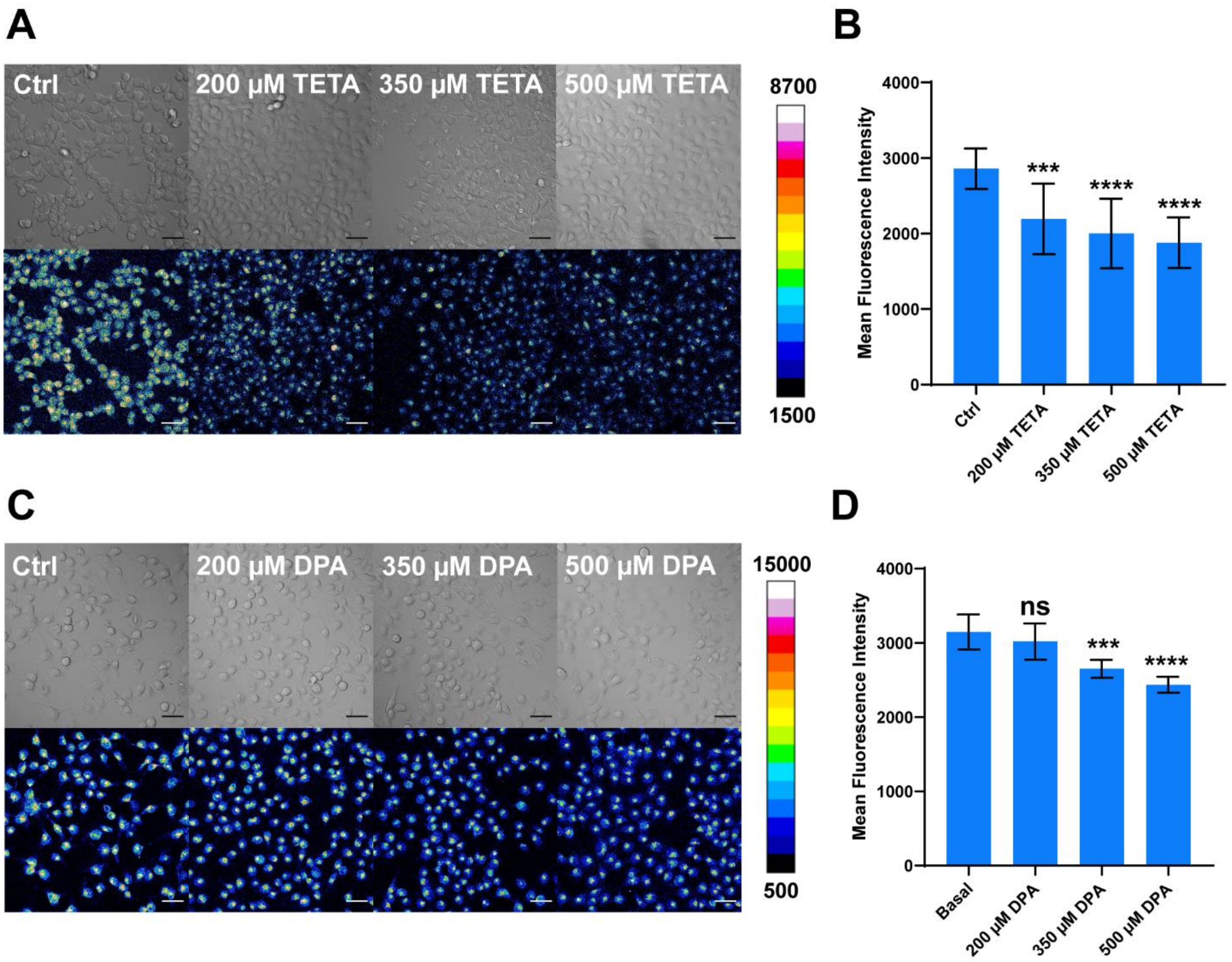

Fig. S15. CD649.2 can detect decreases in endogenous labile Cu(II) in H1299 cells in response to treatment with Cu(II) chelator. Confocal fluorescence microscopy images of H1299 cells incubated with 200-500 µM TETA (A), 200-500 µM DPA (C), or vehicle control in RMPI/10% FBS medium with 1% DMSO for 24 hours. Cells were then washed twice with HBSS (+Ca, Mg), followed by incubation with 1 µM **CD649.2** in HBSS (+Ca, Mg) with 2% DMSO for 30 minutes. Cells were washed once with HBSS (+Ca, Mg) and imaged. Normalized cellular fluorescence intensities of the H1299 cells as determined using ImageJ, showing a dose-dependent decrease in fluorescence signal in response to increasing concentration of TETA (B) or DPA (D). Fluorescence intensity of **CD649.2** was determined from experiments performed in triplicate with λ_{ex} = 633 nm. Error bars denote standard deviation (SD; n = 12). Scale bar = 50 µm. *$p < 0.05$, **$p < 0.01$, ***$p < 0.001$, and ****$p < 0.0001$; ns, not statistically significant.

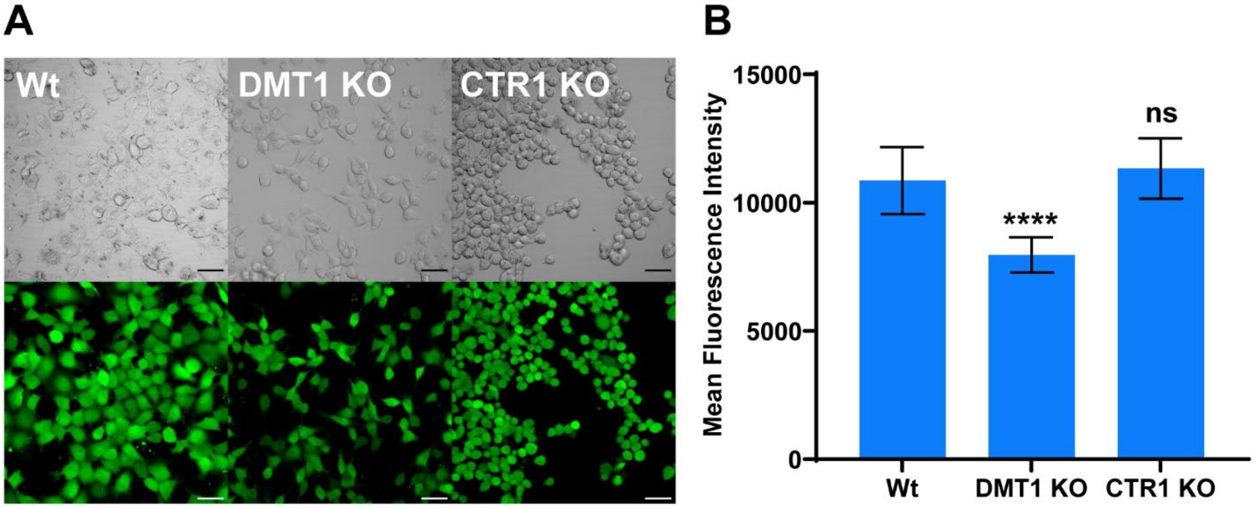

Fig. S16. CD517.2 identifies that DMT1 is a Cu(II)-selective importer. (A) Confocal fluorescence microscopy images of H1299 WT, DMT1 KO, and CTR1 KO cells stained with **CD517.2**. Cells were washed once with HBSS (+Ca, Mg), incubated with 1 μM **CD517.2** in HBSS (+Ca, Mg) for 30 minutes and then imaged. (C) Average cellular fluorescence intensity of **CD517.2** determined using imageJ, showing a decrease in fluorescence signal for DMT1 KO vs. WT and no significant change in signal for CTR1 KO vs. WT. Fluorescence intensity of **CD517.2** was determined from experiments performed in triplicate with λex = 488 nm. Error bars denote standard deviation (SD; n = 12). Scale bar = 50 μm. *$p < 0.05$, **$p < 0.01$, ***$p < 0.001$, and ****$p < 0.0001$; ns, not statistically significant.

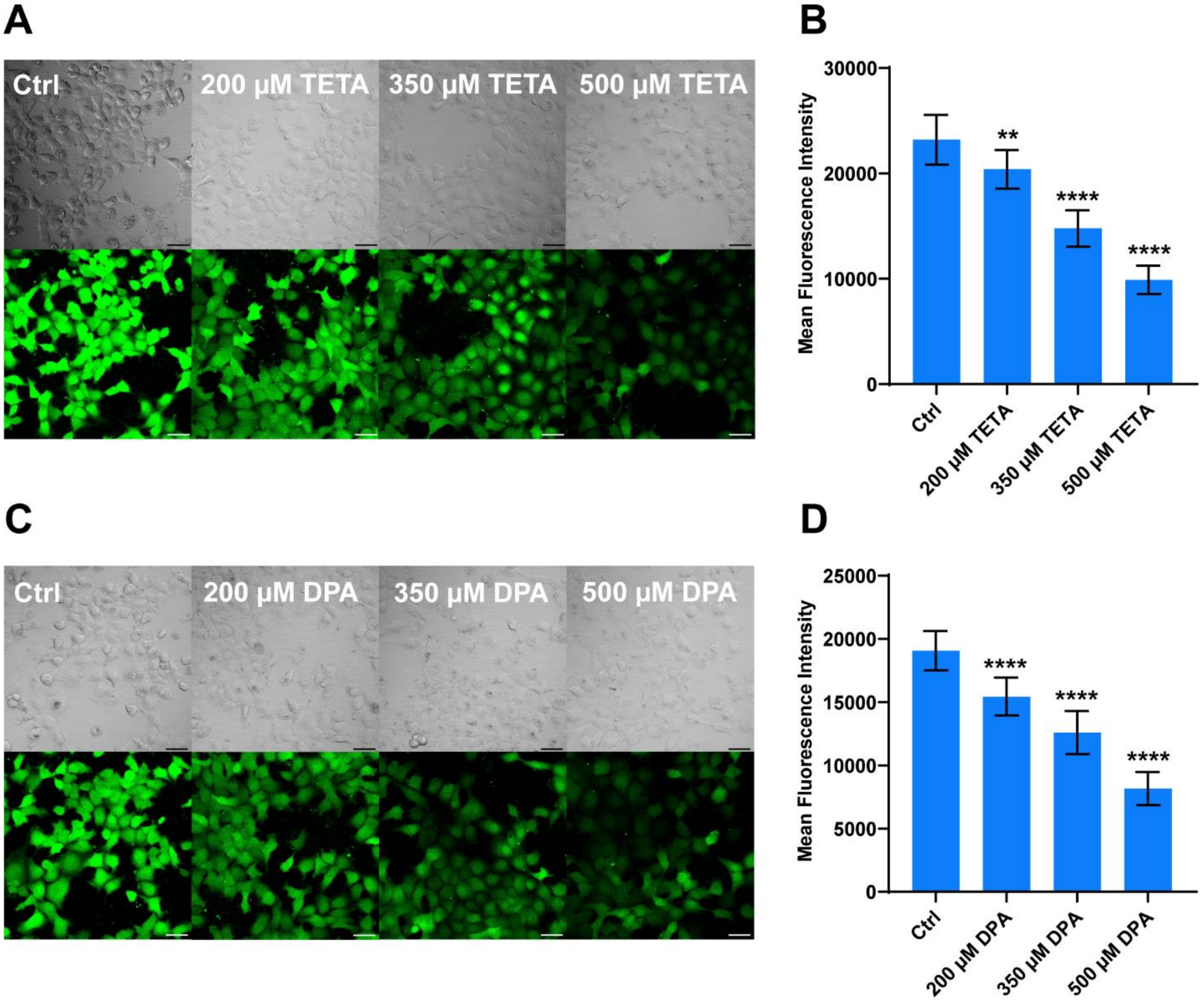

Fig. S17. CD517.2 can detect decreases in endogenous labile Cu(II) in H1299 cells in response to treatment with TETA or DPA. Confocal fluorescence microscopy images of H1299 cells incubated with 200-500 μM TETA (A), 200-500 μM DPA (C), or vehicle control in RMPI/10% FBS medium with 1% DMSO for 24 hours. Cells were then washed twice with HBSS (+Ca, Mg), followed by incubation with 1 μM **CD517.2** in HBSS (+Ca, Mg) with 2% DMSO for 30 minutes. Cells were washed once with HBSS (+Ca, Mg) and imaged. Normalized cellular fluorescence intensities of the H1299 cells as determined using ImageJ, showing a dose-dependent decrease in fluorescence signal in response to increasing concentration of TETA (B) or DPA (D). Fluorescence intensity of **CD649.2** was determined from experiments performed in triplicate with λex = 633 nm. Error bars denote standard deviation (SD; n = 12). Scale bar = 50 μm. $^{*}p < 0.05$, $^{**}p < 0.01$, $^{***}p < 0.001$, and $^{****}p < 0.0001$; ns, not statistically significant.

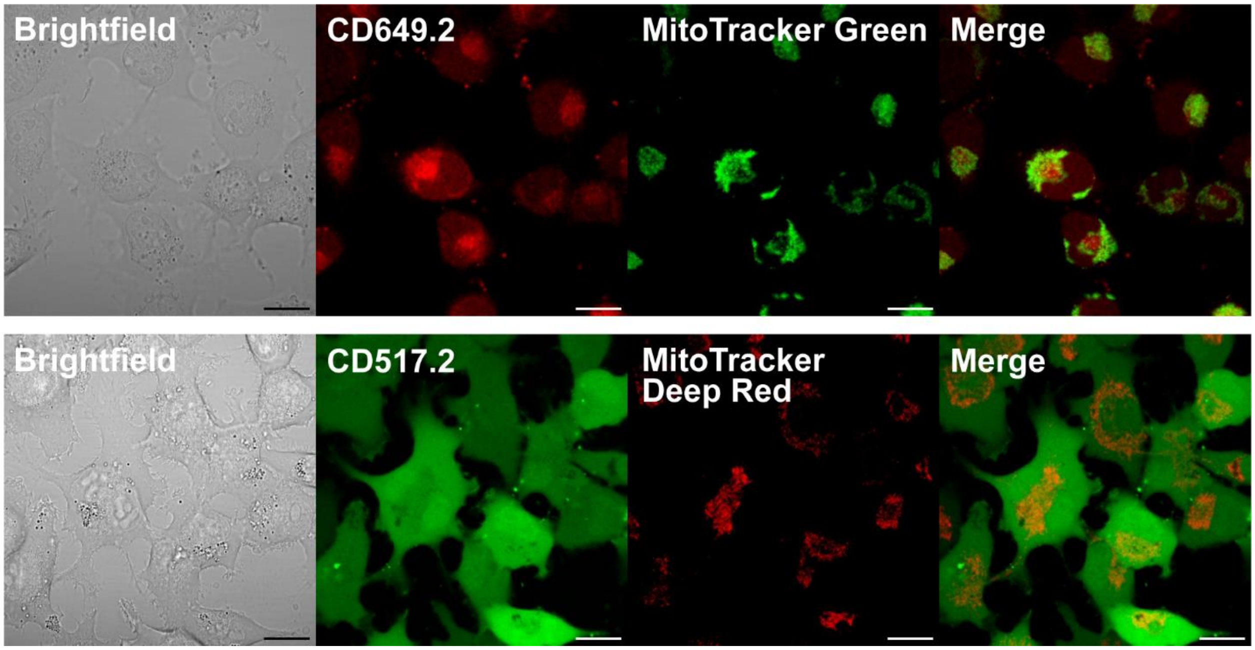

Fig. S18. Confocal fluorescence microscopy images of H1299 cells stained with **CD649.2** and MitoTracker Green or **CD517.2** and MitoTracker Deep Red. Cells were washed once with HBSS (+Ca, Mg), co-incubated with 1 µM **CD649.2** or **CD517.2**, and 250 nM Mito Tracker Green/Deep Red in HBSS (+Ca, Mg) with 2% DMSO for 30 minutes, washed once with HBSS, and then imaged. Merged fluorescence reveals strong co-localization of both probes. **CD649.2** and MitoTracker Deep Red were excited with λex = 633 nm while **CD517.2 and** MitoTracker Green was excited with λex = 488 nm. Scale bar = 20 µm.

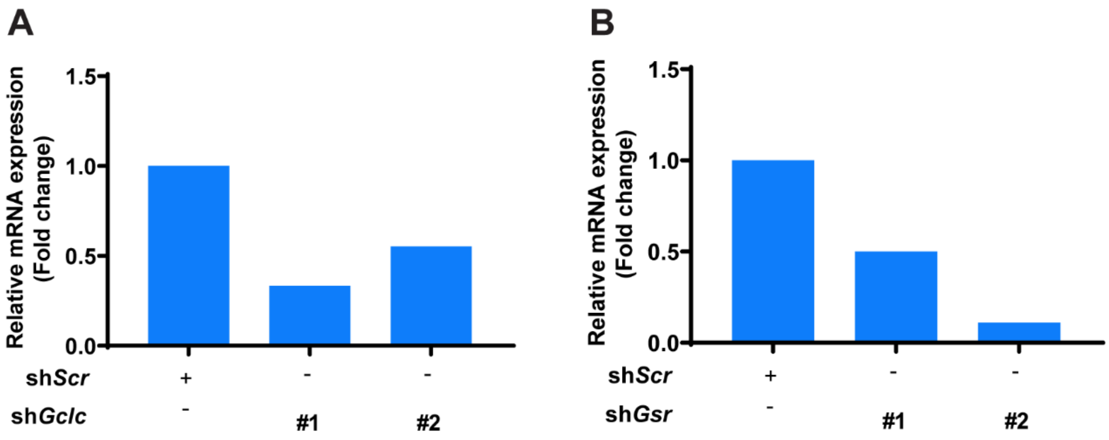

Fig. S19. Normalized qPCR expression of Gclc (A) and Gsr (B) mRNA in mouse embryonic fibroblast (MEF) cells stably expressing nontargeting control shRNA (*Scr*), *Gclc* shRNA, and *Gsr* ShRNA.

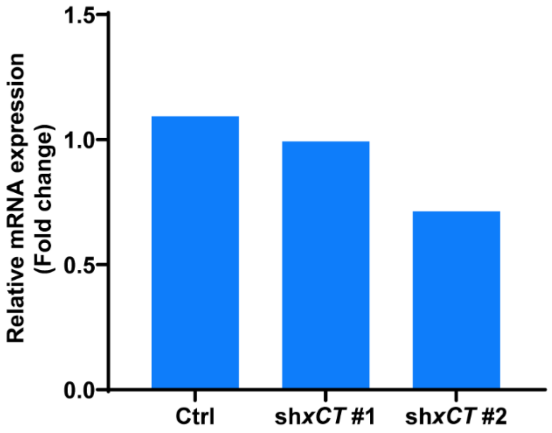

Fig. S20. Normalized qPCR expression of xCT mRNA in mouse embryonic fibroblast (MEF) cells stably expressing different xCT targeting shRNAs, compared to the noninfected parental line (Ctrl).

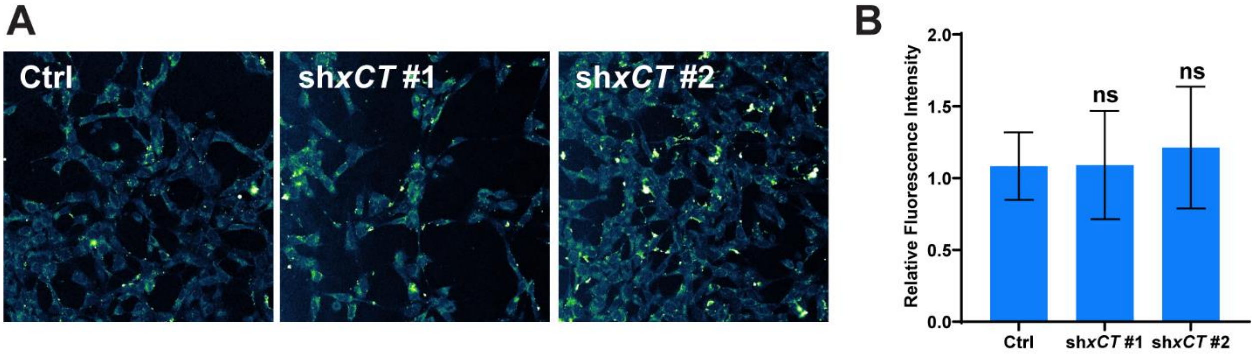

Fig. S21. Live-cell imaging with **CD649.2** reveals unchanged levels of labile Cu(II) pools upon genetic manipulation of the xCT antiporter. (A) Confocal fluorescence images of parental mouse embryonic fibroblast (MEF) cells (Ctrl) and lines stably expressing different shRNAs targeting xCT. Cells were washed twice with HBSS, incubated with 1uM CD649.2 for 1 h, washed with HBSS, and then imaged. (B) Normalized mean CD649.2 fluorescence intensity of MEF cells showing no significant change in signal for both shxCT. Error bars denote standard deviation (SD; n = 3). Fluorescence intensity of CD649.2 was determined from experiments performed in triplicate with λex = 633 nm. $^{*}p < 0.05$, $^{**}p < 0.01$, $^{***}p < 0.001$, and $^{****}p < 0.0001$; ns, not statistically significant.

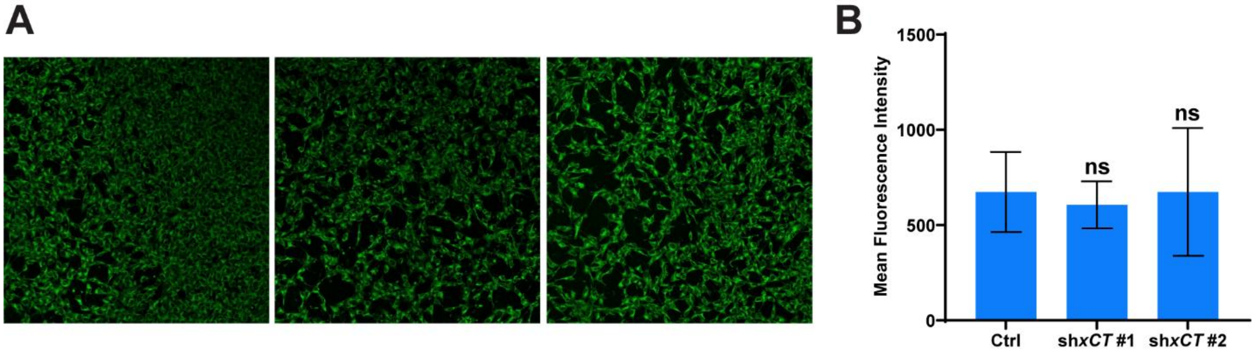

Fig. S22. Live-cell imaging with **CF4** reveals unchanged levels of labile Cu(I) pools upon genetic manipulation of the xCT antiporter. (A) Confocal fluorescence images of parental mouse embryonic fibroblast (MEF) cells (Ctrl) and lines stably expressing different shRNAs targeting xCT. Cells were washed twice with HBSS, incubated with 1 uM CF4 for 20 min, washed with HBSS, and then imaged. (B) Mean CF4 fluorescence intensity of MEF cells showing no significant change in signal for both shxCT. Error bars denote standard deviation Error bars denote standard deviation (SD; n = 2). Fluorescence intensity of CD649.2 was determined from experiments performed in triplicate with $\lambda ex = 514$ nm. *p < 0.05, **p < 0.01, ***p < 0.001, and ****p < 0.0001; ns, not statistically significant.

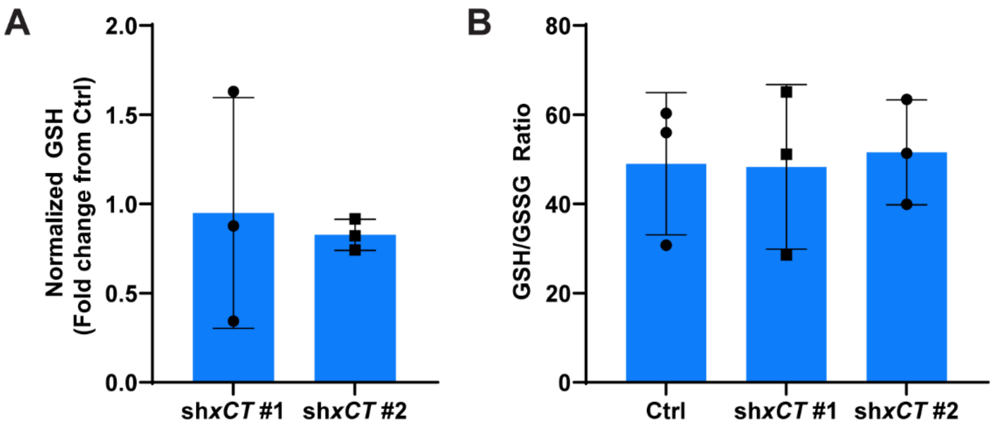

Fig. S23. Quantification of total cellular glutathione (A) or the ratio of GSH to GSSG (GSH/GSSG) (B) ± SD from MEFs stably expressing different xCT shRNAs. The GSH and GSH/GSSG levels were determined using GSH/GSSG-Glo Assay kit, and the results were analyzed using single sample t-tests or 1-way ANOVA, respectively (n=3).

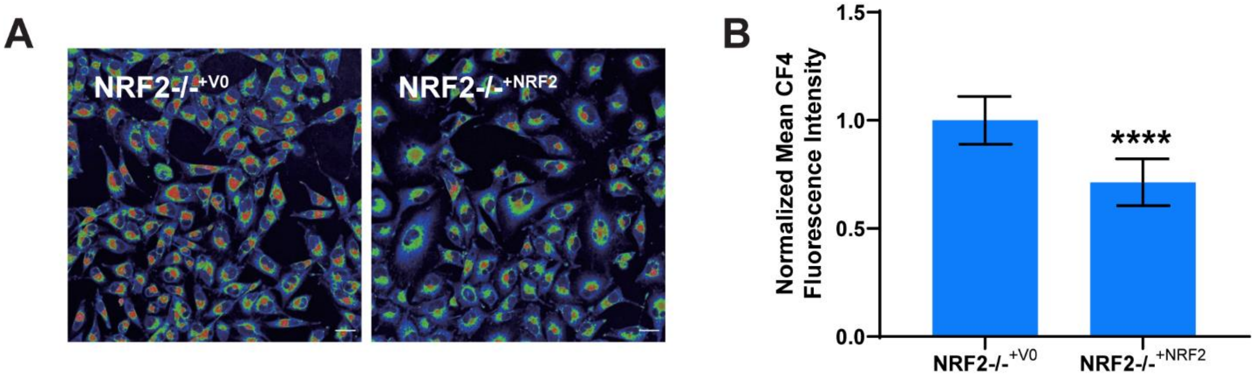

Fig. S24. Live-cell imaging using Copper Fluor-4 (**CF4**) reveals A549 cells lacking NRF2 have higher levels of labile Cu(I). (A) Confocal fluorescence microscopy images of A549 cells stably expressing *NRF2*$^{-/-+V0}$ or *NRF2*$^{-/-+NRF2}$. Cells were washed twice with PBS buffer, incubated with 1 μM **CF4** in Live Cell Imaging Solution for 20 minutes and then imaged. (B) Normalized mean **CF4** fluorescence intensity of A549 cells showing a decrease in signal for *NRF2*$^{-/-+NRF2}$ vs. *NRF2*$^{-/-+V0}$. Error bars denote standard deviation (SD; n = 30). Fluorescence intensity of **CF4** was determined from experiments performed in triplicate. **CF4** was excited by a 458 nm Ar laser and emission was collected at 521 nm. Scale bar = 30 μm. *$p < 0.05$, **$p < 0.01$, ***$p < 0.001$, and ****$p < 0.0001$; ns, not statistically significant.

Synthesis and Characterization Data

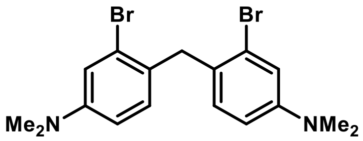

4,4'-Methylenebis(3-bromo-*N*,*N*-dimethylaniline). To a flame-dried round-bottom flask charged with a stirbar was added 3-bromo-*N*,*N*-dimethylaniline (10 g, 50 mmol, 7.1 mL), acetic acid (70 mL) and formaldehyde solution (37 wt% in H_2O, 7.6 mL, 25 mmol). The solution was heated to 60 ˚C and stirred for 2 h. After cooling to room temperature, volatiles were removed with rotary evaporation. Saturated $NaHCO_3$ solution (ca. 150 mL) was added until CO_2 evolution had ceased. The solution was extracted 4x50 mL CH_2Cl_2 and the combined organic layers were washed with H_2O, brine, then dried with Na_2SO_4, filtered, and concentrated. The red residue was recrystallized with hot EtOH to yield a faintly pink powder (6.6 g, 64% yield). The ^{1}H NMR spectrum matches literature reported data (2). ^{1}H NMR (300 MHz, $CDCl_3$) δ 6.96 (s, 2H), 6.85 (d, *J* = 8.5 Hz, 2H), 6.61 (d, *J* = 8.4 Hz, 2H), 4.00 (s, 2H), 2.92 (s, 12H).

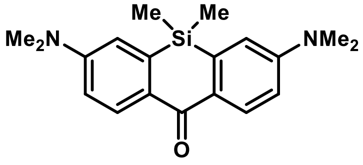

3,7-Bis(dimethylamino)-5,5-dimethyldibenzo[*b,e*]silin-10(5*H*)-one. A flame-dried round-bottom flask charged with a stirbar and 4,4'-methylenebis(3-bromo-N,N-dimethylaniline) (2.20 g, 5.34 mmol) was evacuated and backfilled with N_2 three times. Anhydrous THF (35 mL) was added and the solution was cooled to –78 ˚C. *n*-BuLi (2.5 M, 5.0 mL, 13.9 mmol) was added dropwise and stirred for 30 min at –78 ˚C. Dichlorodimethylsilane (0.84 mL, 6.94 mmol) was added dropwise at –78 ˚C, and the mixture was slowly warmed to room temperature while stirring overnight (ca. 16 h). The reaction was quenched with 15 mL 2 N HCl and subsequently neutralized with saturated $NaHCO_3$ solution. The aqueous phased was extracted 3x with CH_2Cl_2 and the combined organic layer was dried with Na_2SO_4, filtered, and concentrated. The crude material was purified using silica gel column chromatography (eluent: 5 → 40% acetone in hexanes) to yield a yellow solid (390 mg, 23% yield). The ^{1}H NMR spectrum matches literature reported data (3). ^{1}H NMR (300 MHz, $CDCl_3$) δ 8.40 (d, *J* = 8.9 Hz, 2H), 6.84 (dd, *J* = 9.0, 2.8 Hz, 2H), 6.79 (d, *J* = 2.8 Hz, 2H), 3.09 (s, 12H), 0.47 (s, 6H).

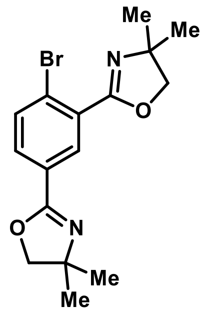

2,2'-(4-Bromo-1,3-phenylene)bis(4,4-dimethyl-4,5-dihydrooxazole). To a flame-dried flask charged with a stirbar and 4-bromoisopthalic acid (5.00 g, 20.4 mmol) was added thionyl chloride (30 mL) and DMF (3 drops). The flask was fitted with a condenser and the reaction was stirred at reflux for 3.5 h. The reaction was then cooled and excess thionyl chloride was removed by vacuum distillation in the fume hood (note: recovered thionyl chloride can be used in the final step of this sequence). The residue was then dissolved in anhydrous CH_2Cl_2 (60 mL). In a separate flame-dried round-bottom flask, 2-amino-2-methyl-1-propanol (5.00 g, 56.1 mmol) was dissolved in anhydrous CH_2Cl_2 (60 mL) and DIPEA (10.0 mL, 57.4 mmol) was added. The former solution was then cannulated into the latter solution and stirred overnight (ca. 16 h) at room temperature. Saturated $NaHCO_3$ solution was added to neutralize the reaction. The mixture was then extracted three times with EtOAc and the combined organic layer was washed with H_2O, brine, then dried with Na_2SO_4, filtered, and concentrated. The residue was then dissolved in thionyl chloride (30 mL) and stirred at room temperature for 1.5 h. Excess thionyl chloride was removed by vacuum distillation in the fume hood. Saturated $NaHCO_3$ solution was added to neutralize the reaction. The mixture was then extracted three times with EtOAc and the combined organic layer was washed with H_2O, brine, then dried with Na_2SO_4, filtered, and concentrated. The crude material was purified using silica gel column chromatography (eluent: 0 → 40% EtOAc in hexanes) to yield a colorless oil (2.16 g, 30% yield over three steps). The ^1H NMR spectrum matches literature reported data (2). ^1H NMR (300 MHz, $CDCl_3$) δ 7.82 (d, J = 2.1 Hz, 1H), 7.43 (dd, J = 8.4, 2.2 Hz, 1H), 7.27 (d, J = 8.4 Hz, 1H), 3.74 (s, 2H), 3.72 (s, 2H), 1.02 (s, 6H), 0.98 (s, 6H).

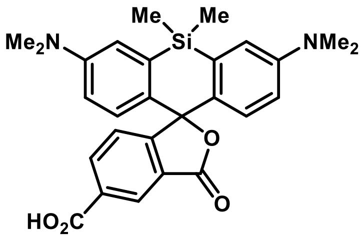

3,7-Bis(dimethylamino)-5,5-dimethyl-3'-oxo-3'*H*,5*H*-spiro[dibenzo[*b,e*]siline-10,1'-isobenzofuran]-5'-carboxylic acid. A flame-dried round-bottom flask charged with a stirbar and 2,2'-(4-bromo-1,3-phenylene)bis(4,4-dimethyl-4,5-dihydrooxazole) (743 mg, 2.12 mmol, 2 equiv.) was evacuated and backfilled with N_2 three times, then anhydrous THF (26 mL) was added and the mixture was cooled to –78 ˚C. *t*-BuLi (1.64 M in pentane, 2.6 mL, 4.3 mmol, 4 equiv.) was added dropwise and stirred for 1 h at –78 ˚C. In another flame-dried round-bottom flask, 3,7-bis(dimethylamino)-5,5-dimethyldibenzo[*b,e*]silin-10(5*H*)-one (343 mg, 1.06 mmol) was dissolved

in anhydrous THF (26 mL) and cooled to –78 ˚C and carefully transferred via cannula to the lithiated solution at –78 ˚C. The reaction was then stirred for 2 h at room temperature after which acetic acid (2 mL) was added to quench the reaction and volatiles were then removed with a rotary evaporator. The residue was then dissolved in 6 N HCl (52 mL) and stirred at 80 ˚C overnight. The reaction was then cooled to room temperature and the pH was adjusted to ~2. The pH was monitored using a pH meter and 6 N NaOH (50 mL) and sat. $NaHCO_3$ solution were used to adjust pH (note: a blue-green precipitate begins to form when nearing pH 2). The suspension was then extracted with 3x50 mL CH_2Cl_2 and the combined organic layers were washed with 0.01 M HCl solution, brine, then dried with Na_2SO_4, filtered and concentrated. The crude material was purified using silica gel column chromatography (eluent: 0 → 5% MeOH in CH_2Cl_2 with 1% acetic acid) to yield a dark blue solid (423 mg, 84% yield). The ^{1}H NMR spectrum matches literature reported data (2). ^{1}H NMR (300 MHz, $CDCl_3$) δ 8.71 (s, 1H), 8.34 (dd, *J* = 8.0, 1.5 Hz, 1H), 7.36 (d, *J* = 8.0 Hz, 1H), 7.17 (d, *J* = 7.5 Hz, 1H), 6.97 (d, *J* = 2.9 Hz, 2H), 6.77 (d, *J* = 8.9 Hz, 2H), 6.57 (dd, *J* = 9.0, 2.9 Hz, 2H), 2.97 (s, 12H), 0.65 (s, 3H), 0.60 (s, 3H).

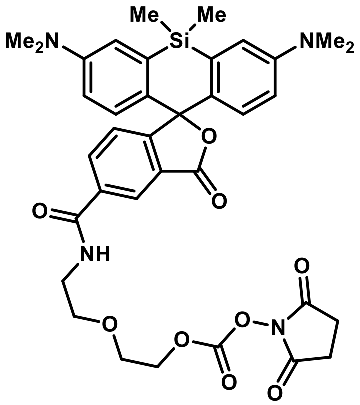

2-(2-(3,7-Bis(dimethylamino)-5,5-dimethyl-3'-oxo-3'*H*,5*H*-spiro[dibenzo[*b,e*]siline-10,1'-isobenzofuran]-5'-carboxamido)ethoxy)ethyl (2,5-dioxopyrrolidin-1-yl) carbonate (2). A flame-dried flask charged with a stirbar, 3,7-bis(dimethylamino)-5,5-dimethyl-3'-oxo-3'*H*,5*H*-spiro[dibenzo[*b,e*]siline-10,1'-isobenzofuran]-5'-carboxylic acid (300 mg, 0.63 mmol) and HATU (292 mg, 0.77 mmol, 1.2 equiv.) was evacuated and backfilled with N_2 three times. Anhydrous CH_2Cl_2 (6 mL) and DIPEA (0.22 mL, 1.3 mmol, 2 equiv) were then added and the stirred together for 30 mins. 2-(2-Aminoethoxy)ethanol (77 µL, 0.77 mmol, 1.2 equiv) was then added and the reaction was stirred at room temperature overnight. Sat. NH_4Cl solution (2 mL) was added to quench the reaction. Additional H_2O was added, and the layer were separated. The aqueous layer was extracted with 3x20 mL EtOAc and the organic layers were combined, washed with brine, dried with Na_2SO_4, filtered and concentrated. A flask containing the residue and N,N′-disuccinimidyl carbonate (DSC, 241 mg, 0.94 mmol, 1.5 equiv.) was evacuated and backfilled with N_2 three times. Anhydrous CH_2Cl_2 (6 mL) and NEt_3 (0.18 mL, 1.3 mmol, 2 equiv) were then added and the reaction was stirred for 48 h at room temperature. Volatiles were removed and the crude mixture was directly purified using silica gel column chromatography (eluent: 50 → 100% EtOAc in hexanes to collect product then 5% MeOH in EtOAc to collect unreacted starting material) to yield a blue-green foamy solid (product: 68 mg, 15% yield; starting material: 100 mg).

N-hydroxysuccinimide (NHS) byproduct coelutes with product to form a 4.5:1 mixture of NHS/product. Product mass and yield have been reported after excluding the mass contribution from NHS. ^{1}H NMR (500 MHz, MeOD) δ 8.37 (s, 1H), 8.20 – 8.16 (m, 1H), 7.32 (d, *J* = 8.1 Hz, 1H), 7.03 (d, *J* = 2.9 Hz, 2H), 6.69 (d, *J* = 9.0 Hz, 2H), 6.60 (dd, *J* = 9.0, 3.0 Hz, 2H), 4.50 – 4.38 (m, 2H), 3.78 – 3.75 (m, 2H), 3.71 (t, *J* = 5.3 Hz, 2H), 3.63 (t, *J* = 5.3 Hz, 2H), 2.94 (s, 12H), 2.73 (s, 4H), 0.63 (s, 3H), 0.55 (s, 3H). *NHS*: 4.59 (s, 1H), 2.80 (s, 4H). ^{13}C NMR (126 MHz, MeOD) δ 172.0, 168.7, 167.5, 158.8, 153.2, 151.1, 138.0, 137.0, 134.6, 132.1, 129.2, 128.1, 126.0, 125.5, 117.8, 114.8, 71.3, 70.5, 69.3, 41.1, 40.5, 29.5, 26.4, 0.4, -1.4. *NHS*: 171.4, 38.9. ESI-MS Calcd. for $C_{36}H_{41}N_4O_9Si^+$ [M+H]$^+$, 701.3; found 701.4 m/z.

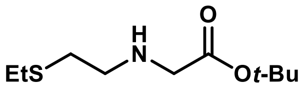

***tert*-Butyl (2-(ethylthio)ethyl)glycinate (3).** To a flame-dried flask charged with a stir bar and 2-(ethylthio)ethylamine (2.2 mL, 20 mmol) was added anhydrous THF (30 mL) and triethylamine (5.6 mL, 40 mmol) and cooled to 0 ˚C. *tert*-Butyl bromoacetate (3.2 mL, 22 mmol) was added dropwise at 0 ˚C and slowly warmed to room temperature stirring overnight (ca. 16 h). Saturated $NaHCO_3$ solution was added, and the mixture was extracted three times with EtOAc. The combined organic layer was dried with Na_2SO_4, filtered, and concentrated. The crude material was purified using silica gel column chromatography (eluent: 30 → 75% EtOAc in hexanes) to yield a pale yellow oil (2.2 g, 51% yield). Alternatively, 2-(ethylthio)ethylamine hydrochloride can be used as starting material with 3.0 equiv. triethylamine to achieve product with a similar yield. The ^{1}H NMR spectrum matches literature reported data (4). ^{1}H NMR (300 MHz, $CDCl_3$) δ 3.31 (s, 2H), 2.79 (td, *J* = 6.5, 1.1 Hz, 2H), 2.66 (td, *J* = 6.5, 1.1 Hz, 2H), 2.54 (q, *J* = 7.4 Hz, 2H), 1.87 (s, 1H), 1.45 (s, 9H), 1.25 (t, *J* = 7.4 Hz, 3H).

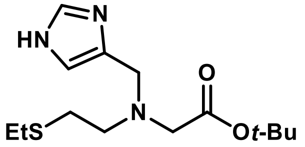

***tert*-Butyl *N*-((1*H*-imidazol-4-yl)methyl)-*N*-(2-(ethylthio)ethyl)glycinate (4).** A solution of **3** (2.00 g, 9.12 mmol), 4-imidazolecarboxaldehyde (1.05 g, 10.9 mmol, 1.2 equiv.) and acetic acid (20 mL) in CH_2Cl_2 (50 mL) was allowed to stir for 30 mins at room temperature. $NaBH(OAc)_3$ (5.17 g, 27.4 mmol, 3 equiv.) was then added and stirring continued for an additional 6.5 h. The reaction was diluted with CH_2Cl_2 (100 mL) and transferred to a separatory funnel, washed with sat. $NaHCO_3$ solution, then brine, then dried with Na_2SO_4, filtered and concentrated. The crude material was purified using silica gel column chromatography (eluent: 5% MeOH in CH_2Cl_2) to yield a waxy, colorless solid (2.16 g, 79% yield). ^{1}H NMR (500 MHz, $CDCl_3$) δ 7.70 – 7.55 (m, 1H), 6.90 (s, 1H), 3.80 (s, 2H), 3.27 (s, 2H), 2.85 (dd, *J* = 8.4, 6.2 Hz, 2H), 2.61 (dd, *J* = 8.4, 6.2 Hz, 2H), 2.48 (q, *J* = 7.4 Hz, 2H), 1.44 (s, 9H), 1.21 (t, *J* = 7.4 Hz, 3H). ^{13}C NMR (126 MHz, $CDCl_3$)

δ 171.1, 135.2, 131.6, 122.6, 81.4, 55.6, 53.5, 49.4, 30.0, 28.3, 26.1, 14.9. ESI-MS Calcd. for $C_{14}H_{26}N_3O_2S^+$ $[M+H]^+$, 300.2; found 300.2 m/z.

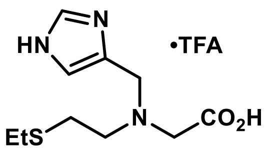

***N*-((1*H*-Imidazol-4-yl)methyl)-*N*-(2-(ethylthio)ethyl)glycine trifluoroacetic acid adduct (5, NSCO$_2$).** The solid compound **4** (2.15 g, 7.18 mmol) was dissolved in CH_2Cl_2 (2 mL) and trifluoroacetic acid (10 mL) then stirred at room temperature overnight. Volatiles were removed with a rotary evaporator and residual trifluoroacetic acid was removed with toluene. The resulting red oil was recrystallized with hexanes/CH_2Cl_2 and a small amount of MeOH to yield a colorless powder (2.36 g, 92% yield). ^{1}H NMR (500 MHz, MeOD) δ 8.85 (s, 1H), 7.64 (s, 1H), 4.31 (s, 2H), 3.80 (s, 2H), 3.17 (dd, J = 8.8, 6.4 Hz, 2H), 2.81 (dd, J = 8.8, 6.2 Hz, 2H), 2.58 (q, J = 7.4 Hz, 2H), 1.26 (t, J = 7.4 Hz, 3H). ^{19}F{^{31}C} NMR (470 MHz, MeOD) δ -77.22. ^{13}C NMR (126 MHz, MeOD) δ 172.0, 162.0 (q, $^2J_{CF}$ = 36.5 Hz), 136.4, 129.7, 120.6, 117.6 (q, $^1J_{CF}$ = 290.0 Hz), 55.0, 54.6, 49.8, 28.5, 26.6, 15.1. ESI-MS Calcd. for $C_{10}H_{18}N_3O_2S^+$ $[M+H]^+$, 244.1; found 244.2 m/z.

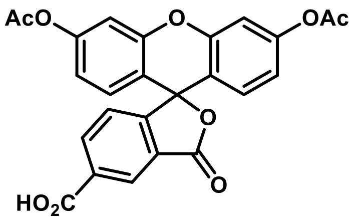

5-Carboxyfluorescein diacetate (S1). The synthesis was carried out according to a literature procedure (5). To a solution of 5-carboxyfluorescein (5.28 g, 14.03 mmol) in pyridine (60 mL, 744.9 mmol) at room temperature, acetic anhydride (36 mL, 380.8 mmol) was added dropwise. After stirring for 20 hours, the reaction mixture was diluted with 200 mL EtOAc to obtain a yellow/brown solution. The organic layer was washed twice with 100 mL 1M NaCO3, once with 100 mL of saturated NH_4Cl solution and 100 mL brine successively, before the organic residue was dried over anhydrous $MgSO_4$ and concentrated in vacuo. The residue was recrystallized twice in CH_2Cl_2 and MeOH, followed by silica gel column chromatography (eluent: 0 → 10% MeOH in CH_2Cl_2 gradient) to afford a white crystalline solid (5.19 g, 80%) . ^{1}H NMR (600 MHz, Methylene Chloride-*d*2) δ 8.77 (s, 1H), 8.46 (dd, J = 8.0, 1.3 Hz, 1H), 7.37 (d, J = 8.0 Hz, 1H), 7.18 (d, 2H), 6.90 (m, 4H), 2.34 (s, 6H). ^{13}C NMR (151 MHz, Methylene Chloride-*d*2) δ 168.79, 167.80, 165.45, 156.77, 152.47, 151.54, 136.69, 132.31, 128.81, 127.22, 126.77, 124.43, 118.04, 115.74, 110.55, 81.75, 20.87. ESI-MS Calcd. for $C_{25}H_{16}O_9$ $[M+H]^+$, 461.4; found 461.4 m/z.

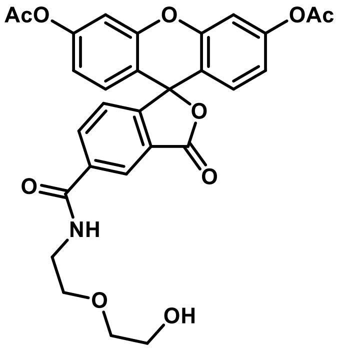

5-((2-(2-Hydroxyethoxy)ethyl)carbamoyl)-3-oxo-3H-spiro[isobenzofuran-1,9'-xanthene]-3',6'-diyl diacetate (S2). Synthesis was performed according to a modifying literature procedure (6). A solution of **S1** (2.12 g, 4.60 mmol, 1.00 equiv.), HATU (2.10 g, 5.52 mmol, 1.2 equiv.) and N,N-Diisopropylethylamine (1.60 ml, 9.21 mmol, 2.00 equiv.) in 15 mL CH_2Cl_2 was added to a 250 mL round bottom flask. After stirring at room temperature for 0.5 hours, 2-(2-aminoethoxy) ethanol (0.484 g, 4.60 mmol, 1.00 equiv.) was added and the reaction mixture was stirred for another 4 hours. 50 mL of saturated NH_4Cl solution was added and the aqueous layer was washed with EtOAc. The organic fractions were dried over anhydrous $MgSO_4$ and concentrated in vacuo. The crude material was purified using silica gel column chromatography (eluent: 100% CH_2Cl_2 → 19/1 CH_2Cl_2/MeOH) yielding a yellow oil. The product was re dissolved in EtOAc and washed thrice with 50 mL of brine, before the aqueous layers were combined and washed once with 20 mL EtOAc. The organic fractions were aggregated, dried over anhydrous $MgSO_4$ and concentrated in vacuo, to reveal a white powder (1.12 g, 44%). ^{1}H NMR (600 MHz, Methylene Chloride-*d*2) δ 8.43 (s, 1H), 8.18 (dd, *J* = 8.0, 1.4 Hz, 1H), 7.27 (d, *J* = 8.0 Hz, 1H), 7.12 (m, 2H), 6.81 (m, 4H), 3.73 (t, 2H), 3.67 (s, 4H), 3.59 (t, 2H), 2.30 (s, 6H). ^{13}C NMR (151 MHz, Methylene Chloride-*d*2) δ 168.92, 168.41, 166.04, 154.84, 152.39, 151.52, 136.85, 134.69, 128.77, 126.48, 124.36, 123.74, 118.02, 115.72, 110.48, 82.01, 72.09, 69.94, 61.46, 40.06, 20.87. ESI-MS Calcd. for $C_{29}H_{25}NO_{10}$ $[M+H]^+$, 548.5; found 548.5 m/z.

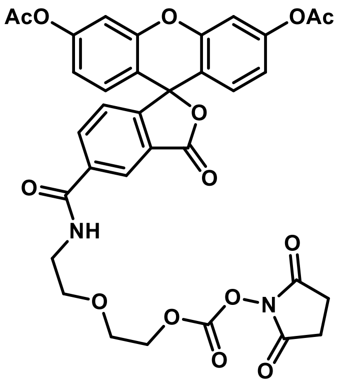

5-((2-(2-((((2,5-Dioxopyrrolidin-1-yl)oxy)carbonyl)oxy)ethoxy)ethyl)carbamoyl)-3-oxo-3H-spiro[isobenzofuran-1,9'-xanthene]-3',6'-diyl diacetate (S3). Synthesis was performed according to a modifying literature procedure (6). A solution of **S2**, (1.00 g, 1.83 mmol, 1.00 equiv.) and triethylamine (0.25 mL, 1.83 mmol, 1.00 equiv.) in 25 mL CH_2Cl_2, and a solution

of N,N′-disuccinimidyl carbonate (DSC, 702 mg, 2.741 mmol, 1.50 equiv.) dissolved in 5 mL CH_2Cl_2 were combined in a 100 mL round bottom flask. The reaction was left to stir at room temperature for 1 hour. Upon addition of triethylamine the solution turned yellow and following the addition of DSC the solution reverted to colourless. The crude material was purified using silica gel column chromatography (eluent: 9/1 CH_2Cl_2/EtOAc to 3/2 CH_2Cl_2/EtOAc) to yield a white powder (1.05 g, 83%). ^{1}H NMR (600 MHz, Methylene Chloride-*d*2) 8.40 (s, 1H), δ 8.25 (d, J = 8.0Hz, 1H), 7.30 (d, J = 8.0 Hz, 1H), 7.24 (s, 1H), 7.13 (m, 2H), 6.81 (m, 4H), 4.53 (t, 2H),3.73 (t, 2H), 3.72 (s, 4H), 2.84 (s, 4 H), 2.31 (s, 6H) ^{13}C NMR (151 MHz, Methylene Chloride-*d*2) δ 169.36, 168.76, 168.28, 165.54, 154.77, 152.37, 151.74, 151.54, 136.95, 134.96, 128.84, 126.44, 124.26, 123.48, 117.95, 115.85, 110.44, 81.79, 69.82, 69.73, 68.21, 40.11, 25.54, 20.83. HR-ESI-MS Calcd. for $C_{34}H_{28}N_2O_{14}$ $[M+H]^+$, 689.1613; found 689.1618 m/z.

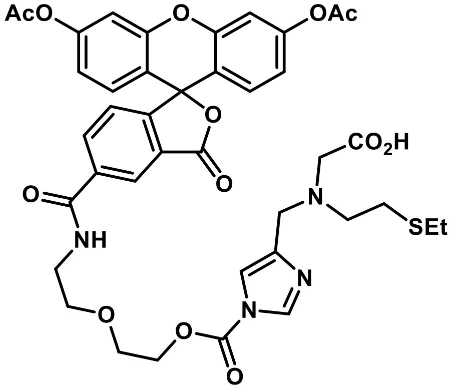

CD517.2. A solution of **5**, (57 mg, 0.234 mmol, 1.50 equiv.) and N,N-Diisopropylethylamine, (0.03 ml, 0.17 mmol, 1.10 equiv.) in 5 mL CH_2Cl_2, and a solution of **S3** (1.00 equiv, 107 mg, 0.156 mmol) dissolved in 3 mL CH_2Cl_2 were combined in a 25 mL round bottom flask. The reaction was left to stir at room temperature for 1 hour. The reaction mixture was concentrated *in vacuo* and the residue was dissolved in 1 mL CH_2Cl_2 and 20 mL of diethyl ether. The resultant mixture was filtered, and the precipitate was collected and dissolved in a minimal amount of DMSO. The crude mixture was then purified by prep HPLC (3/7 MeOH/H_2O → 100% MeOH) to yield a white solid (8 mg, 6.3%). ^{1}H NMR (600 MHz, Methylene Chloride-*d2*) δ 8.42 (s, 1H) 8.21 (d, J = 7.9 Hz, 1H), 8.12 (s, 1H), 7.46 (s, 1H), 7.31 (d, J = 8.0 Hz, 1H), 7.14 (s, 2H), 6.85 (m, 4H), 4.58 (m, 2H), 3.86 (m, 2H), 3.77 (s, 2H), 3.69-3.75 (m, 4H), 3.36 (s, 2H), 2.90 (t, J = 6.8 Hz, 2H), 2.69 (t, J = 6.9 Hz, 2H), 2.50 (q, J = 7.3 Hz, 2H), 2.31 (s, 6H), 1.21 (t, J = 7.3 Hz, 3H). ^{13}C NMR (151 MHz, Methylene Chloride-*d2*) δ 168.75, 168.18, 165.37, 165.35, 154.89, 152.40, 151.55, 148.50, 139.11, 137.37, 136.97, 134.72, 128.81, 126.57, 124.41, 123.32, 117.98, 117.94, 115.80, 110.47, 81.83, 69.62, 68.50, 67.16, 56.45, 54.05, 50.87, 39.97, 29.65, 25.83, 20.83, 14.49. HR-ESI-MS Calcd. for $C_{40}H_{40}N_4O_{13}S$ $[M+H]^+$, 817.2385 and $[M+Na]^+$, 839.2205; found 817.2388 m/z and 839.2201 m/z.

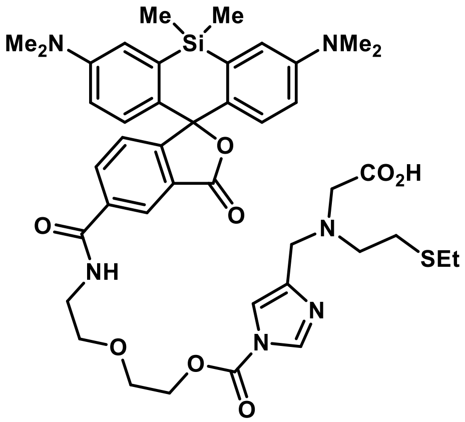

CD649.2. A flame-dried round-bottom flask charged with a stirbar, **2** (57 mg, 8.2 µmol, *NHS mass contribution excluded),* and **5** (80 mg, 23 µmol) was evacuated and backfilled with N_2 three times. Anhydrous DMF (4 mL) and triethylamine (59 µL, 42 µmol) were added and the reaction was stirred at room temperature for 48 h. Volatiles were removed and the crude mixture was directly purified using silica gel column chromatography (eluent: 5:4:1 → 9:0:1 → 85:0:15 CH_2Cl_2/CH_3CN/MeOH with 1% acetic acid) to yield a blue-green foamy solid (product: 66 mg, 97% yield). ^{1}H NMR (500 MHz, $CDCl_3$) δ 8.43 (s, 1H), 8.27 (d, *J* = 8.0 Hz, 1H), 8.14 (s, 1H), 7.46 (s, 1H), 7.39 (d, *J* = 8.0 Hz, 1H), 6.96 (d, *J* = 2.8 Hz, 2H), 6.69 (d, *J* = 8.9 Hz, 2H), 6.50 (dd, *J* = 9.0, 2.8 Hz, 2H), 4.53 (s, 2H), 3.83 – 3.68 (m, 8H), 3.33 (s, 2H), 3.05 – 2.83 (m, 14H), 2.67 (t, *J* = 7.3 Hz, 2H), 2.46 (q, *J* = 7.4 Hz, 2H), 1.17 (t, *J* = 7.4 Hz, 3H), 0.64 (s, 3H), 0.59 (s, 3H). ^{13}C NMR (126 MHz, $CDCl_3$) δ 170.2, 166.2, 156.9, 149.5, 148.6, 137.7, 137.4, 135.6, 133.9, 131.1, 128.3, 127.6, 125.4, 123.7, 116.8, 116.4, 113.3, 92.8, 69.9, 68.6, 67.3, 56.8, 54.3, 51.0, 45.2, 40.3, 40.1, 28.6, 26.1, 14.8, 8.8, 0.6, -1.6. ESI-MS Calcd. for $C_{42}H_{53}N_6O_8SSi^+$ $[M+H]^+$, 829.3; found 829.5 m/z.

References

1. C. J. K. Wijekoon, T. R. Young, A. G. Wedd, Z. Xiao, CopC Protein from Pseudomonas fluorescens SBW25 Features a Conserved Novel High-Affinity Cu(II) Binding Site. *Inorg. Chem.* **54**, 2950–2959 (2015).
2. R. Wirth, P. Gao, G. U. Nienhaus, M. Sunbul, A. Jäschke, SiRA: A Silicon Rhodamine-Binding Aptamer for Live-Cell Super-Resolution RNA Imaging. *J. Am. Chem. Soc.* **141**, 7562–7571 (2019).
3. R. Sato, *et al.*, Intracellular Protein-Labeling Probes for Multicolor Single-Molecule Imaging of Immune Receptor–Adaptor Molecular Dynamics. *J. Am. Chem. Soc.* **139**, 17397–17404 (2017).
4. E. L. Que, *et al.*, Copper-Responsive Magnetic Resonance Imaging Contrast Agents. *J. Am. Chem. Soc.* **131**, 8527–8536 (2009).
5. J. Pan, *Transition Metal Catalyzed Cyclization and Synthesis of Triptolide Analogs* (BiblioBazaar, 2017).
6. T. Miki, *et al.*, A conditional proteomics approach to identify proteins involved in zinc homeostasis. *Nat. Methods* **13**, 931–937 (2016).
